# Conformation and dynamics of the kinase domain drive subcellular location and activation of LRRK2

**DOI:** 10.1073/pnas.2100844118

**Published:** 2021-06-04

**Authors:** Sven H. Schmidt, Jui-Hung Weng, Phillip C. Aoto, Daniela Boassa, Sebastian Mathea, Steve Silletti, Junru Hu, Maximilian Wallbott, Elizabeth A. Komives, Stefan Knapp, Friedrich W. Herberg, Susan S. Taylor

**Affiliations:** ^a^Department of Biochemistry, University of Kassel, 34132 Kassel, Germany;; ^b^Department of Pharmacology, University of California, San Diego, La Jolla, CA 92093;; ^c^Department of Chemistry and Biochemistry, University of California, San Diego, La Jolla, CA 92093;; ^d^National Center for Microscopy and Imaging Research, University of California, San Diego, La Jolla, CA 92093;; ^e^Department of Neurosciences, University of California, San Diego, La Jolla, CA 92093;; ^f^Institute for Pharmaceutical Chemistry, Johann Wolfgang Goethe-University, D-60438 Frankfurt am Main, Germany;; ^g^Structural Genomics Consortium, Buchmann Institute for Molecular Life Sciences, Johann Wolfgang Goethe-University, D-60438 Frankfurt am Main, Germany

**Keywords:** leucine-rich repeat kinase 2 (LRRK2), hydrogen-deuterium exchange mass spectrometry (HDX-MS), Gaussian accelerated molecular dynamics, kinase regulation, Parkinson’s disease

## Abstract

To explore how pathogenic mutations of the multidomain leucine-rich repeat kinase 2 (LRRK2) hijack its finely tuned activation process and drive Parkinson’s disease (PD), we used a multitiered approach. Most mutations mimic Rab-mediated activation by “unleashing” kinase activity, and many, like the kinase inhibitor MLi-2, trap LRRK2 onto microtubules. Here we mimic activation by simply deleting the inhibitory N-terminal domains and then characterize conformational changes induced by MLi-2 and PD mutations. After confirming that LRRK2_RCKW_ retains full kinase activity, we used hydrogen-deuterium exchange mass spectrometry to capture breathing dynamics in the presence and absence of MLi-2. Solvent-accessible regions throughout the entire protein are reduced by MLi-2 binding. With molecular dynamics simulations, we created a dynamic portrait of LRRK2_RCKW_ and demonstrate the consequences of kinase domain mutations. Although all domains contribute to regulating kinase activity, the kinase domain, driven by the DYGψ motif, is the allosteric hub that drives LRRK2 regulation.

## Significance

To achieve a mechanistic understanding of LRRK2, a multidomain protein kinase, we must understand how the conformational landscape is changed by specific mutations that cause LRRK2 to become a driver of Parkinson’s disease (PD). To meet this challenge, we used a construct, LRRK2_RCKW_, that lacks the N-terminal inhibitory domains. Both catalytic domains as well as full activity are retained in LRRK2_RCKW_. To capture solvent-exposed/protected regions, we used hydrogen-deuterium exchange mass spectrometry and showed in detail how the conformation changed in the presence of a kinase inhibitor, MLi-2. Using molecular dynamics simulations, we explored the effects of MLi-2 as well as PD mutations on dynamics. Our multitiered analysis defines the kinase domain as a dynamic allosteric hub for LRRK2 activation.

**Fig. 1.**
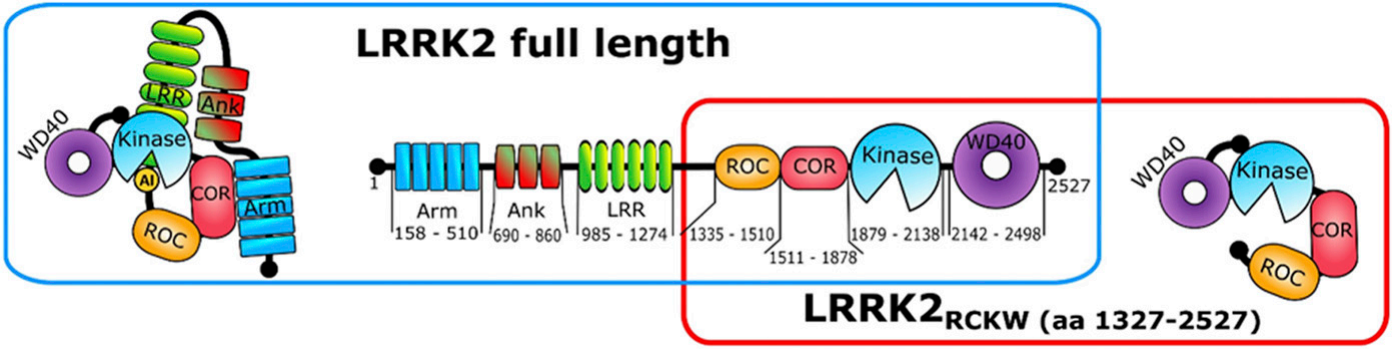
Schematic domain organization of LRRK2. Full-length protein (blue box) consists of the Armadillo domain (Arm), Ankyrin repeat (Ank), leucine-rich repeat, a GTPase domain called Ras of complex, C terminus of the Roc domain, kinase domain, and WD40 domain. The N-terminal domains contain the Arm, Ank, and LRR domains. The C-terminal domains, corresponding to the LRRK2_RCKW_ construct (red box), contain the ROC, COR, kinase, and WD40 domains.

Mutations within leucine-rich repeat kinase 2 (LRRK2) are the most common cause for genetically driven forms of Parkinson’s disease (PD) (1). LRRK2 is a complex and multifunctional protein consisting of seven closely interacting structural domains (Fig. 1) (2). A special feature of LRRK2 is that it contains both a kinase domain and a GTPase (Ras of complex; ROC) domain. The kinases and the GTPases are the two most important switches in biology, so LRRK2 provides a special opportunity to explore these two catalytically active domains embedded in the same polypeptide chain (3).

Familial PD mutations in LRRK2 lead to altered cellular phenotypes such as microtubule (MT)-associated filament formation, impeded vesicular trafficking, as well as changes in nuclear morphology (4–7). Some of these PD mutations such as G2019S in the kinase domain lead to increased kinase activity while others do not (7–9), so what actually drives PD is thus still ambiguous, although it is clear that the kinase domain plays an important pathogenic role. Two of the four most common PD mutations, G2019S and I2020T, are located in the highly conserved DFGψ motif, which in LRRK2 is DYGI. The importance of this motif was further supported by our earlier discovery that Tyr2018 in this motif, conserved as Phe in most other protein kinases, is a critical part of the activation switch mechanism (10). With five scaffolding and two catalytically active domains, the interdomain cross-talk and how these domains effect and regulate each other is very complex. However, due to the lack of high-resolution structural data not much was known until recently about these interactions and how they intrinsically regulate LRRK2. LRRK2 dimerization, and interactions with other proteins such as Rabs and 14-3-3 proteins, further increase the complexity of LRRK2 regulation (11–16).

To unravel this complexity, we used a multitiered approach beginning with a cell-based assay for filament formation, a process that correlates with LRRK2 docking onto MTs (6). While these highly decorated MTs are not likely found in patients, they display a feature that is likely to be a transient state in the physiological life cycle of LRRK2 as it toggles between its active and inactive conformations; PD mutations simply shift the equilibrium and trap LRRK2 in a “frozen” state. Specifically, we used live-cell imaging to examine the reversible spatial and temporal distribution of full-length wild-type (WT) and G2019S LRRK2 in HEK293T cells in the presence and absence of LRRK2 kinase inhibitors. To discriminate between the regulatory functions that are embedded in the catalytically inert N-terminal domains (NTDs) and the catalytic functions that are in the C-terminal domains (CTDs), we engineered a construct, LRRK2_RCKW_, that contains only the CTDs (ROC, COR [C terminus of the ROC domain], kinase, and WD40 domains). This construct contains both catalytic moieties and is the smallest fragment that is capable of docking onto MTs (5). In contrast to full-length LRRK2 and the G2019S mutant, the corresponding LRRK2_RCKW_ constructs formed filaments spontaneously in the absence of MLi-2, a high-affinity kinase inhibitor. To confirm that the catalytic machinery is conserved in LRRK2_RCKW_, we characterized the kinase activity of WT and mutant LRRK2_RCKW_ proteins using LRRKtide and Rab8a as substrates. We focused, in particular, on the DYGI motif in the kinase domain which is a hotspot for PD mutations and a critical part of the switch mechanism that leads to LRRK2 activation (10). Using hydrogen-deuterium exchange mass spectrometry (HDX-MS) to map the conformational state of LRRK2_RCKW_, we obtained a comprehensive profile of the solvent-accessible and -protected regions confirming this large multidomain protein is well-folded. We next mapped changes in the solvent exposure of the LRRK2_RCKW_ domains following binding of MLi-2. A detailed analysis of these changes mapped onto a model of the active kinase domain provided a comprehensive allosteric portrait of the kinase domain as a hub for driving long-range conformational changes. At a final level, to create a dynamic portrait of LRRK2_RCKW_, we used Gaussian accelerated molecular dynamics (GaMD) simulations to observe in silico at an atomistic level how single-amino acid mutations in the kinase domain contribute to the intrinsic dynamic regulation of LRRK2.

Our multiscale approach allowed us to achieve a deeper appreciation of the intrinsic molecular features of LRRK2 and emphasized the crucial role of the DYGI motif in regulating LRRK2 structure and function. We hypothesized that the NTDs of LRRK2 (ARM, ANK, and LRR) play an inhibitory regulatory role acting as a “lid,” which is “unleashed” physiologically by activated Rab proteins but also by some of the PD mutations. Experimentally, the NTDs can be unleashed from the CTDs by kinase inhibitors or by simply deleting the NTDs, leaving the CTDs catalytically intact. Strikingly, our resulting LRRK2 model for activation and subcellular localization closely resembles the activation process of Raf kinases, which further supports our model and the central concept of kinases serving as the hub for driving conformational switching in multidomain signaling proteins.

## Results

To characterize and dissect the functional properties of the catalytic domains of LRRK2, in particular the kinase domain, we used a multiscale approach that extends from testing real-time filament formation in live cells to assessing the consequences of MD simulations of PD mutations in the kinase domain. Of primary importance initially was to characterize the biochemical properties of LRRK2_RCKW_ following deletion of the catalytically inert NTDs. To next confirm that LRRK2_RCKW_ was a well-folded protein, we used HDX-MS. To capture the allosteric features of the kinase domain, we mapped by HDX-MS the conformational changes in LRRK2_RCKW_ that result from adding MLi-2. Finally, we explored single-site mutants in silico using MD simulations.

### Capturing Filament Formation in Real Time.

**Fig. 2.**
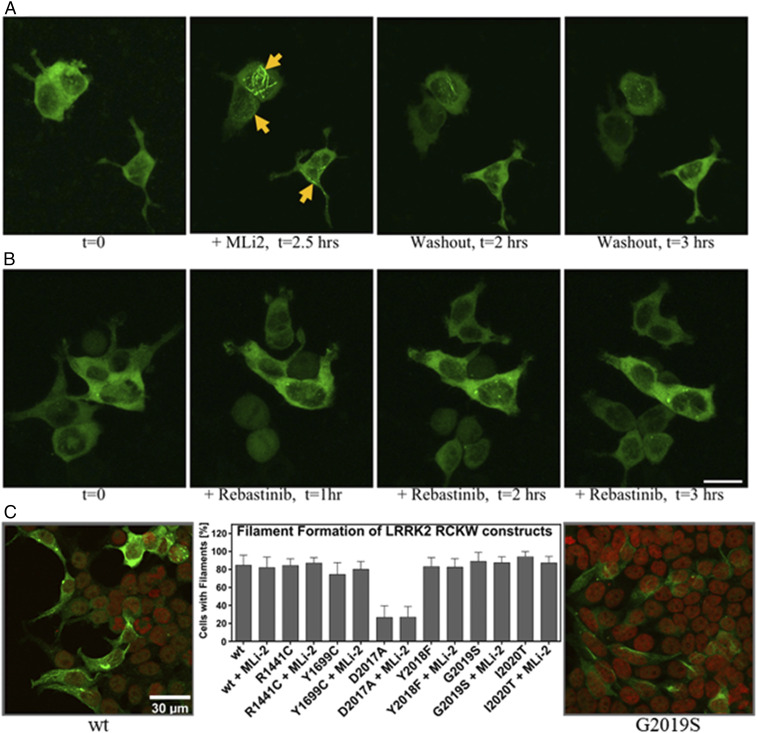
Localization of the LRRK2-G2019S mutant and LRRK2_RCKW_ variants. (*A*) Time-lapse imaging of HEK293T cells transiently expressing YFP-LRRK2-G2019S: Confocal images (YFP fluorescence signal, maximum-intensity projections) were acquired every 11 min. Representative images show the typical diffuse cellular localization of the proteins (*t* = 0 h) prior to treatment with 100 nM MLi-2; following MLi-2 addition, proteins relocalize to form cytoplasmic filamentous structures (yellow arrows; +MLi-2, *t* = 2.5 h). After washout of the inhibitor, the proteins gradually dissociate from the filaments into the cytosol (washout; *t* = 2 to 3 h). (*B*) Time-lapse imaging of HEK293T cells transiently expressing YFP-LRRK2-G2019S before (*t* = 0 h) and after treatment with 100 nM rebastinib. No changes in the localization of the proteins are observed. (Scale bar, 20 μm.) (*C*) Plasmids, encoding LRRK2_RCKW_ variants, were transfected into HEK293T cells for LRRK2_RCKW_ overexpression. Transfected cells were then analyzed for the spatial distribution of LRRK2_RCKW_ by immunostaining. All tested LRRK2_RCKW_ variants displayed a high likelihood (80 to 90%) of forming filaments inside the HEK293T cells except for LRRK2_RCKW_ D2017A (20 to 30%). Interestingly, in contrast to full-length LRRK2, the percentage of cells showing filament formation was independent of MLi-2 treatment or a specific LRRK2_RCKW_ mutation. The figures show the filaments of WT and G2019S. The figures of other LRRK2_RCKW_ variants are shown in *SI Appendix*, Fig. S2. Error bars represent standard deviations (SD) for the percentage of filament forming cells on six to ten representative images taken per transfection (*n* = 2).

We and others previously showed that treatment with a highly specific LRRK2 inhibitor (MLi-2) induces filament formation of WT LRRK2 and the G2019S mutant when these proteins are transiently expressed in mammalian cells (7, 10). To capture the dynamics of such redistribution, we performed time-lapse imaging of yellow fluorescent protein (YFP)-tagged G2019S and WT LRRK2 (G2019S: Movie S1; WT: Movie S3). As shown in Fig. 2 and Movies S1, S2, S3, and S4, under normal conditions G2019S LRRK2 is mostly diffuse in the cytosol; however, 15 to 30 min following MLi-2 treatment the protein begins to concentrate first in “satellite” structures diffuse throughout the cells. It then polymerizes to form intricate thicker filaments by 2.0 to 2.5 h after treatment. Although WT LRRK2 follows a similar redistribution upon treatment with MLi-2, in general it takes longer, ∼30 min to 1 h, before the first structures are observed (Movies S1, S2, S3, and S4). In both cases this effect is readily reversible: After washout of MLi-2 for 2 h, the proteins gradually diffuse back into the cytosol (G2019S: Movie S2; WT: Movie S4). To verify that this protein rearrangement was truly dependent on the specific MLi-2 inhibitor, we performed time-lapse imaging using a type 2 inhibitor, rebastinib (Fig. 2*A*). Although rebastinib tightly binds to the LRRK2 kinase domain, as established from the stabilization of a kinase–WD40 construct in a thermal shift assay (*SI Appendix*, Fig. S1), it did not induce changes in the localization of G2019S proteins even after 8-h treatment, confirming the prediction of Deniston et al. (5).

### LRRK2_RCKW_ Variants Spontaneously Form Filaments around Microtubules in an MLi-2–Independent Manner.

In our filament formation assay, Flag-tagged variants of the LRRK2_RCKW_ construct were overexpressed and cells were analyzed after fixation via antibody staining by a confocal laser-scanning microscope. The majority of the transfected cells, regardless of the mutation, displayed constitutive filament formation (*SI Appendix*, Fig. S2). Most striking, in contrast to full-length LRRK2, is that WT and G2019S are no longer dependent on MLi-2 for docking onto MTs (Fig. 2*C*). This supports the hypothesis that the inert NTDs are not required for the filaments to form but instead are essential for protecting or shielding the catalytic domains to prevent them from docking onto MTs. In this way, they promote the cytosolic distribution of LRRK2 prior to activation, which is likely further facilitated by phospho-dependent interactions with specific 14-3-3 proteins (11, 15). The N-terminal domains are also important for docking to Rab proteins such as Rab29, which is thought to initiate activation of LRRK2 (14), and to Rab substrates such as Rab8a and Rab10. This provides a physiological mechanism where multiple biological functions are embedded in the catalytically inert N-terminal domains, including activation and/or localization by heterologous proteins as well as inhibition of the catalytic domains. We hypothesize that most of the PD mutations use different mechanisms to circumvent or “hijack” this normal process.

Of the mutants tested, only LRRK2_RCKW_ D2017A, a kinase-dead mutant, showed strongly reduced docking onto MTs, which is consistent with our earlier findings showing that the full-length D2017A mutant did not dock onto MTs even in the presence of MLi-2 (*SI Appendix*, Fig. S2). We confirmed here that MLi-2 did not have an additive effect on the percentage of cells showing LRRK2_RCKW_ filaments and did not induce binding of the D2017A mutant (*SI Appendix*, Fig. S2). We conclude that the high-affinity binding of MLi-2 to the kinase domain is sufficient to unleash the N-terminal protective lid that normally shields the catalytic domains and promotes localization in the cytosol. We also show that simply removing the N-terminal lid is in most cases sufficient to promote docking onto MTs. The exception is the D2017A mutant, which cannot bind well under any conditions either because it lacks the ability to undergo a subsequent essential autophosphorylation step or, most likely, because it is locked into an open conformation similar to what we saw with rebastinib. We next asked whether LRRK2_RCKW_ retained its full kinase catalytic activity even though the regulatory machinery embedded in the N-terminal domains is removed.

### Protein Kinase Activity Is Conserved in the LRRK2_RCKW_ Proteins.

**Fig. 3.**
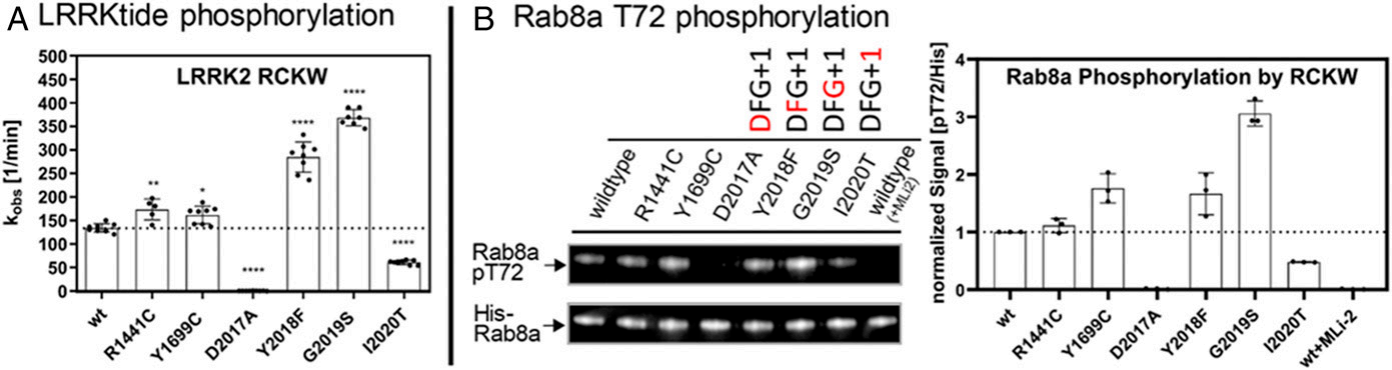
LRRK2_RCKW_ variants Y2018F and G2019S enhance LRRKtide and Rab8a phosphorylation. (*A*) An LRRKtide-based kinase assay for LRRK2_RCKW_ variants revealed that it preserves full-length LRRK2 kinase activity. Additionally, the DYGψ mutants tested here also resemble the results of their full-length counterparts. Interestingly, also the pathogenic mutations R1441C and Y1699C which are situated in the ROC–COR region of the LRRK2_RCKW_ construct display a mild increase in kinase activity compared with LRRK2_RCKW_ WT. Asterisks indicate the *P* value by one-way ANOVA test: 0.01 < *P < 0.05; 0.001 < **P < 0.01; ****P < 0.0001. Error bars represent SD for at least five independent measurements. (*B*) When testing Rab8a as a substrate for the LRRK2_RCKW_ construct employing Western blotting against pT72 and the His tag of His-Rab8a, we revealed increased phosphorylation of Rab8a by LRRK2_RCKW_ Y2018F, G2019S, and Y1699C. MLi-2 was shown to efficiently block phosphorylation of Rab8a which was also found for the kinase-dead mutant D2017A. Quantification was performed for three independent Western blots. For each quantification, the pT72 signals were referenced to the signal for the His tag of 6×His-Rab8a and then normalized to the resulting WT signal. The dotted line therefore represents 100% of the WT signal. Error bars represent SD of the quantification of three independent Western blots.

To assess kinase activity, we used both LRRKtide, a small synthetic peptide, and Rab8a as substrates for the LRRK2_RCKW_ proteins. In addition to WT LRRK2_RCKW_, we measured the kinase activities of two ROC–COR domain mutations (R1441C and Y1699C) and four mutations in the kinase domain, more precisely in the DYGψ motif (D2017A, Y2018F, G2019S, and I2020T). R1441 and Y1699 are located in the ROC and COR domains, respectively, and, based on homology models, are predicted to be part of the ROC–COR domain interface (5, 17, 18). Importantly, we found that WT LRRK2_RCKW_ has kinase activity that is comparable to full-length LRRK2 (10) although in the absence of the N-terminal scaffolding domains the activity is no longer dependent on Rab activation. Using LRRKtide as a substrate, we found that the pathogenic mutation R1441C slightly increased the kinase activity while Y1699C had only a minor effect on LRRKtide phosphorylation (Fig. 3). In contrast, when we used a physiological substrate, Rab8a, Y1699C led to an enhanced pT72 phosphorylation in vitro, comparable to the phosphorylation by Y2018F, whereas R1441C behaved like WT (Fig. 3). The fact that kinase activity is dependent on substrate may account for some of the confusion in the literature about the activity of various LRRK2 mutants and suggests that some of the mutations may simply change substrate specificity. If these residues are indeed at a domain interface, as predicted, they could also introduce a conformational change that would result in the unleashing of the N-terminal scaffolding domains and/or promote dimerization which is associated with membrane localization and activation of LRRK2 (14, 19).

The strongest effects on kinase activity for LRRK2_RCKW_ were observed for mutations embedded within the activation segment of the kinase domain, specifically in the DYGI motif, where ψ is typically conserved as a hydrophobic residue. The Tyr is a Phe in most other kinases, and Y2018 was predicted earlier, based on activation when the Tyr is replaced with Phe, to serve as a brake that keeps LRRK2 in an inactive state (10). We measured the effect of mutating each of these residues on kinase activity. The D2017A (DYG) mutant was not able to phosphorylate either LRRKtide or Rab8a (Fig. 3) which is consistent with other kinases, since this residue is part of the regulatory triad and is crucial for the correct coordination of the Mg^2+^ ions and the γ-phosphate of adenosine triphosphate (ATP) in the kinase active-site cleft (20). Reintroducing the classical DFGψ motif to LRRK2_RCKW_ increases the kinase activity for LRRKtide by a factor of 3 to 4, whereas Rab8a phosphorylation was only enhanced by a factor of 1.7 (Fig. 3). LRRKtide phosphorylation by LRRK2_RCKW_ G2019S was comparable to LRRK2_RCKW_ Y2018F. When comparing Rab8a pT72 phosphorylation, G2019S showed two times higher Rab8a pT72 phosphorylation than Y2018F. The other tested pathogenic DYGψ mutation, I2020T, displayed a reduced phosphorylation of LRRKtide as well as Rab8a (Fig. 3). This is also in accordance with our full-length LRRK2 data of I2020T, albeit full-length I2020T Rab8a phosphorylation was comparable to WT. The results for the I2020T mutation in full-length LRRK2 and LRRK2_RCKW_ demonstrate that LRRK2 pathogenicity is not driven solely by increased kinase activity but also by changed substrate preferences such as serine/threonine specificity as well as changes in subcellular localization. We later show that the dynamic properties are also altered by these mutations.

### Mapping the Conformational Changes Induced by MLi-2 Using Hydrogen-Deuterium Exchange Mass Spectrometry.

To define the global conformational changes induced in LRRK2_RCKW_ as a consequence of MLi-2 binding, we used HDX-MS, which allows us to determine the solvent-exposed regions of the protein over a time course of 5 min. Although this is a large protein (1,200 residues), we obtained excellent coverage (>96%), and the solvent-exposed regions are consistent with the predicted folding of all four domains (*SI Appendix*, Fig. S3). While we focus here primarily on the kinase domain, the graph summarizing the overall solvent accessibility of the entire protein shows not only that the four domains are well-folded but also identifies several regions that are highly solvent-exposed. Of particular note is the activation loop of the kinase domain as well as the segment that lies between the COR-B domain and the kinase domain and the segment that joins the GTPase domain to the COR-A domain. The HDX-MS data suggest that these regions having high deuterium uptake are highly flexible or unfolded. Conversely, there are also regions on the surface of each domain that are highly protected from solvent, implying that these are domain–domain interfacial surfaces (*SI Appendix*, Fig. S3). It is important to appreciate that the HDX-MS profile is obtained independent of a solved structure and can thus serve as validation of a predicted model. Overall, LRRK2_RCKW_ is a well-folded protein that is consistent with a complex topological model with interdomain interactions.

#### Kinase domain.

**Fig. 4.**
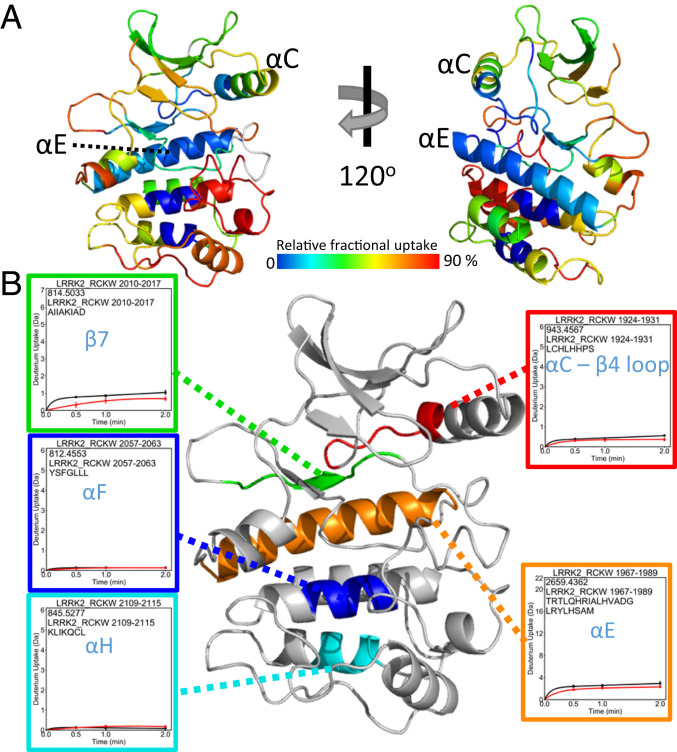
Deuterium uptake of the LRRK2_RCKW_ kinase domain. (*A*) The relative deuterium uptake after 2 min of deuterium exposure of the LRRK2_RCKW_ kinase domain is shown in a color-coded homology model. Gray color indicates no deuterium uptake information. The N lobe of the kinase mostly shows blue to green colors indicating low deuterium uptake. On the other hand, the αD-helix, activation loop, and end of αF-helix to αH-helix have higher deuterium uptake suggesting a more dynamic, solvent-accessible C lobe. (*B*) Representative peptides that have almost no deuterium uptake are mapped on the kinase domain. (*B*, *Insets*) Uptake for the apo kinase (black) and the MLi-2–bound state (red).

Under apo conditions the N lobe of the kinase domain is more shielded from solvent than the C lobe (Fig. 4*A*). The αC-β4-loop, for example, is almost completely shielded from solvent. This is somewhat unusual in that the N lobe in the absence of nucleotide tends to be rather dynamic for many protein kinases. The ordered and stable structure of the N lobe of the kinase domain is predicted to be due to constraints imposed by the flanking domains. This is analogous to the way that cyclin binding orders the N lobe of CDK2 in contrast to the isolated kinase domain (21). Most kinase structures represent just an isolated kinase domain, so one cannot appreciate how other domains contribute to stabilization and, in turn, regulation of the N lobe. Our HDX-MS results also help to explain why it has not been possible so far to express the kinase domain independent of the rest of the LRRK2_RCKW_ domains. For example, deletion of the ROC domain or deletion of even a few residues at the C terminus abolish the kinase activity (6).

In the apo protein the activation loop of the kinase domain in the C lobe has the highest deuterium uptake, suggesting it is highly disordered and exposed to solvent. In contrast to the activation loop, the helices of the C lobe for the most part are highly protected (Fig. 4*B*). The αE-helix, for example, is completely shielded from solvent, as is the middle of the αF-helix and the C terminus of the αH-helix. β7 is packed against a portion of the αC-β4-loop, and these two peptides that include the Mg-positioning loop and the last turn of the αC-helix are also completely shielded from solvent. These shielded regions nicely define the hydrophobic core of the kinase domain (Fig. 4*B*).

#### Effect of inhibitor binding.

**Fig. 5.**
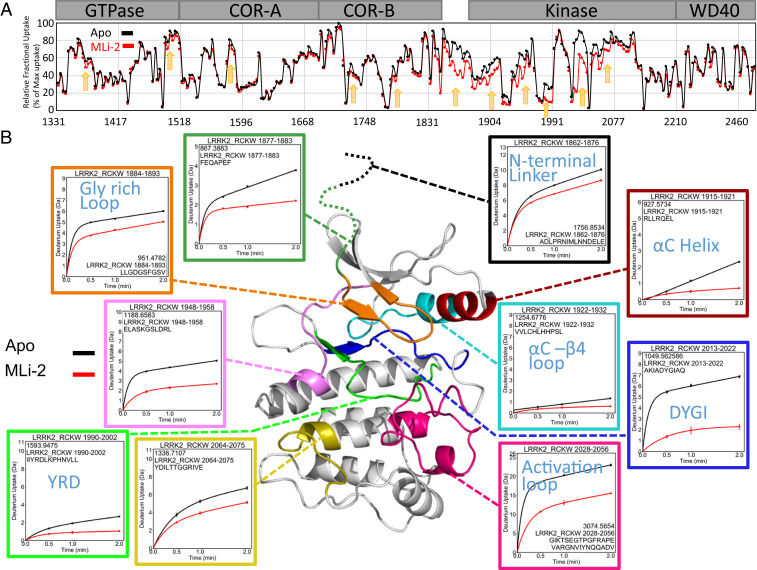
Binding of MLi-2 reduces the deuterium uptake of LRRK2_RCKW_. (*A*) The relative deuterium exchange for each peptide detected from the N to C terminus of LRRK2_RCKW_ in apo kinase (black) and MLi-2–bound (red) conditions at 2 min. The arrows indicate the regions of LRRK2 that have less deuterium uptake when bound to MLi-2. (*B*) The deuterium uptake of selected peptides is plotted and mapped on the kinase model. The uptake is reduced in the Gly-rich loop, αC-helix, activation loop, DYGI motif, YRD motif, and hinge region in the presence of MLi-2.

To gain insight into the allosteric impact of inhibitor binding, we next looked at the conformational changes in LRRK2_RCKW_ following treatment with MLi-2. The overall changes, captured in the graph in Fig. 5*A*, show that there is subtle, albeit important, protection in regions that extend into the GTPase and COR-A–COR-B domains; however, the largest changes are concentrated in the kinase domain and in the linker that precedes the kinase domain. We focus here on the conformational changes that are localized to our kinase domain model. These changes lie not only in the N lobe and the active-site cleft but also in the C lobe in regions that lie far from the active-site cleft.

Among the protected regions, we saw that the binding of MLi-2 reduces the H-D exchange in the ATP-binding site, activation loop, αC-helix, and hinge region (Fig. 5*B*). These regions that would be predicted to contact the inhibitor (22) all show significantly reduced deuterium uptake. Peptides, for example, in the hinge region (amino acids 1948 to 1958), including the αD-helix, experienced a large increase in protection upon MLi-2 binding (50 vs. 20%). The peptide covering the catalytic loop (amino acids 2013 to 2022) including the YRD motif also experienced protection (30 to <10%), and the glycine-rich loop (amino acids 1884 to 1893) is also highly protected. Most importantly, we see that the peptide containing the DYGI motif (amino acids 1990 to 2002) is almost completely shielded as a consequence of MLi-2 binding; the deuterium exchange dropped from 70 to less than 10%, suggesting that this region, highly solvent-accessible in the absence of ligand, becomes almost completely protected by the coordination of the inhibitor. This is quite consistent with the prediction that the kinase domain assumes a compact and closed conformation in the presence of MLi-2. The C terminus of this peptide contains the beginning of the activation loop, which now also appears to be well-folded and shielded from solvent in contrast to the apo structure.

**Fig. 6.**
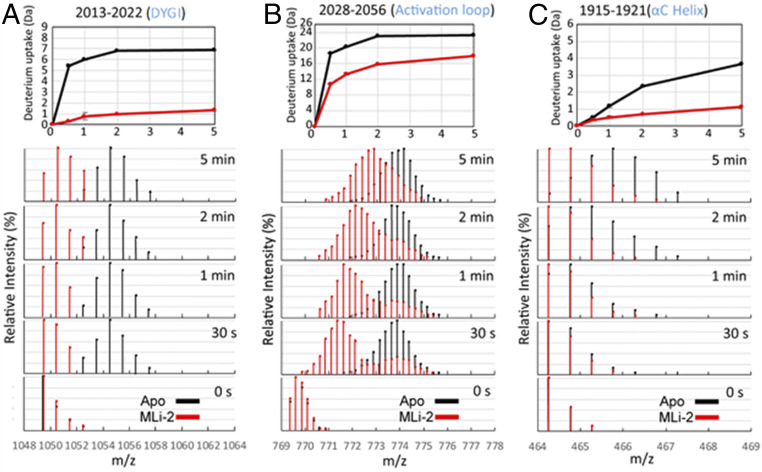
Deuterium uptake and spectral plot of peptides in the DYGI (*A*), activation loop (*B*), and αC-helix (*C*) reveal slow dynamics. In the DYGI peptide (amino acids 2013 to 2022) the apo state (black) plateaus within 2 min. The MLi-2–bound state (red) continues to slowly exchange at least up to 5 min, suggesting that with MLi-2 this region undergoes a slow dynamic process. The apo state of the activation-loop peptide (amino acids 2028 to 2056) again plateaus within 2 min while the MLi-2–bound state gradually increases after 2 min. From the spectral plot, the uptake of the activation-loop peptide in the MLi-2 state exhibits bimodal behavior. One process has slow deuterium uptake (protected) and the other process has fast uptake (solvent-exposed)—similar to the single process observed in the apo state. For the αC-peptide (amino acids 1915 to 1921), the deuterium increases without reaching a plateau over 5 min for both states.

We looked more closely at the dynamic features of some of the critical peptides in the C lobe (Fig. 6). Of particular interest are the uptake spectra of the two peptides covering the activation loop: Both show an EX1 bimodal distribution, a feature that is indicative of two different conformations in solution (23). One of these peptides (amino acids 2028 to 2056) is shown in Figs. 5*B* and 6*B*. All other peptides, such as the DYGI peptide shown in Fig. 6*A*, show a single peak indicative of the more typical EX2 exchange kinetics. In addition, MLi-2 treatment also induced slow exchange in the DYGI loop even though it is highly protected. The peptide that covers the N terminus of the αC-helix (amino acids 1915 to 1921) shows significant slow exchange even in the absence of MLi-2 that most likely continues beyond 5 min (Fig. 6*C*). Although the exchange is quenched in the presence of MLi-2, the slow exchange still persists.

Although the protection of the ATP-binding site and the hinge region by MLi-2 is consistent with other inhibitor-bound homolog kinase structures (22, 24), the detailed information revealed by HDX-MS without structure is remarkable. In addition, our data also reflect the dynamic change that the binding of MLi-2 has not only on the kinase domain but also on LRRK2_RCKW_ overall. Essentially any region in LRRK2_RCKW_ that interfaces with the kinase domain will sense binding of nucleotide or an inhibitor. This also includes the NTDs, not included in our construct, which are predicted to lie over the kinase domain and inhibit kinase activity when LRRK2 is in its inactive state (5, 25). As demonstrated earlier, the NTDs would be displaced by the high-affinity binding of a kinase inhibitor. HDX-MS shows specifically how changes in conformation and dynamics of the kinase domain are felt through long distances in LRRK2_RCKW_, as flexible regions throughout the protein exhibit increased protection upon MLi-2 binding (Fig. 5*A* and *SI Appendix*, Fig. S3). This protection will be a good predictor of domain interfaces that change as a consequence of MLi-2 binding.

### GaMD Simulations Indicate That the LRRK2 Kinase Domain Mutations Y2018F, G2019S, and I2020T Attenuate Flexibility of the Activation Segment of the Kinase Core.

**Fig. 7.**
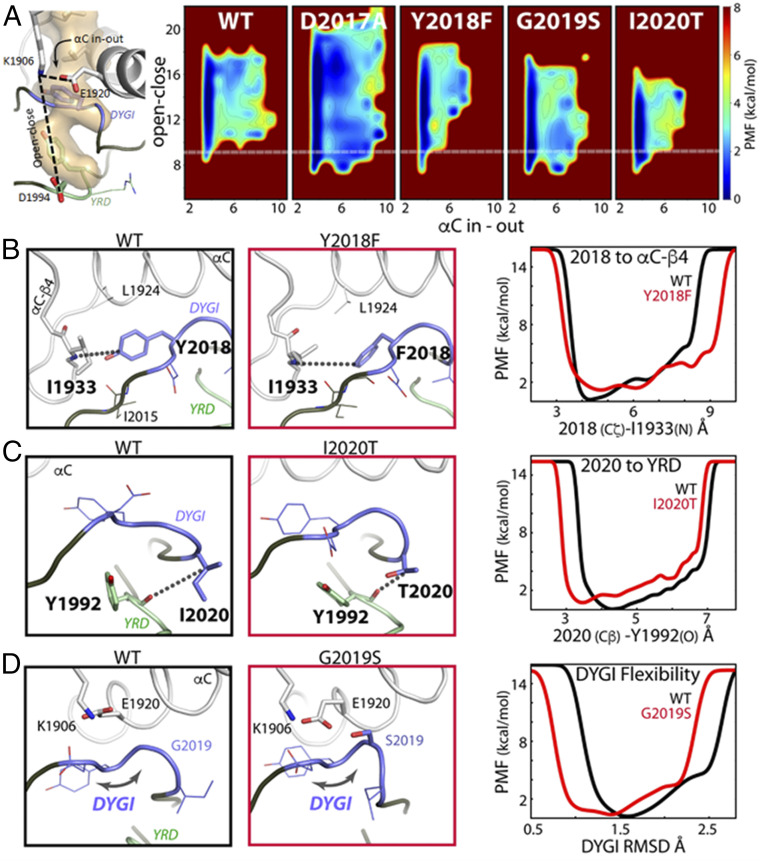
Mutations in the DYGψ loop alter kinase dynamics. (*A*) Kinase conformational free-energy landscape, represented by “open–close”: the distance from the top (K1906/β3-sheet) to the bottom (D1994/YRD motif) of the active site; and “αC in–out”: the distance between K1906 and E1920/αC-helix. The white line shows the closed-active kinase conformation. WT samples the active state infrequently, whereas the mutants more readily access the closed-active conformation. However, D2017A is destabilized to a more open conformation. PMF, potential of mean force. (*B*) In WT, Y2018 is locked in an inactive orientation by hydrogen bonds with I2015 and I1933. Y2018F packs with L1924 and releases the DYGI loop from an inactive state helping to assemble the active site. Y2018F breaks the interaction leading to increased side-chain dynamics, measured by the distance between the 2018 ζ-carbon and the backbone of I1933. (*C*) I2020T makes a hydrogen bond with the backbone of Y1992, coupling the DYGI and catalytic loops, which decreased backbone dynamics. The mutation brings the DYGI and YRD motifs together, measured as the distance from the 2020 Cβ and the backbone of Y1992. (*D*) G2019S bridges the DYGI loop to the αC-helix and β3-sheet, through E1920 and K1906. This stabilizes the DYGI loop, shown by rmsd, and promotes the closed kinase conformation.

GaMD simulations were performed on the activated kinase domain of LRRK2 (amino acids 1865 to 2135) to investigate changes in the conformational landscape that are caused by the D2017A, Y2018F, G2019S, and I2020T mutations. Enhanced sampling was used to broadly sample conformational space in order to build an accurate representative model of the LRRK2 kinase and the changes induced by mutation. During all 10 replicate accelerated simulations the WT kinase favors an open and inactive active-site cleft conformation as measured by the relative positions of the N and C lobes and the αC-helix (Fig. 7). The fully closed and active conformation, in which the N and C lobes are brought together in concert with an inward positioning of the αC-helix to assemble the active site, is infrequently sampled by the WT kinase. In contrast, Y2018F, G2019S, and I2020T are all capable of accessing a closed and active conformation, while D2017A samples a much more open and inactive conformation (Fig. 7*A*). The degree of stabilization of the closed conformation roughly correlates with the observed changes in MT association: D2017A < WT < G2019S < Y2018F < I2020T; it does not correlate with activity. The I2020T mutant is trapped in a mostly closed state without extensive open-to-close transitions and αC in-to-out motion compared with WT, Y2018F, and G2019S. This loss of breathing dynamics may partially explain the reduced kinase activity of I2020T, where substrate/product kinetics may be impacted. Likewise, Y2018F and G2019S both populate a wide range of open and also closed active conformations likely contributing to their increased kinase activity. The ability of all of the activating mutants to populate a closed conformation may play a role in their altered MT association compared with WT, where Y2018F and I2020T spontaneously form filaments and G2019S forms filaments faster than WT upon treatment by the type I inhibitor MLi-2.

**Fig. 8.**
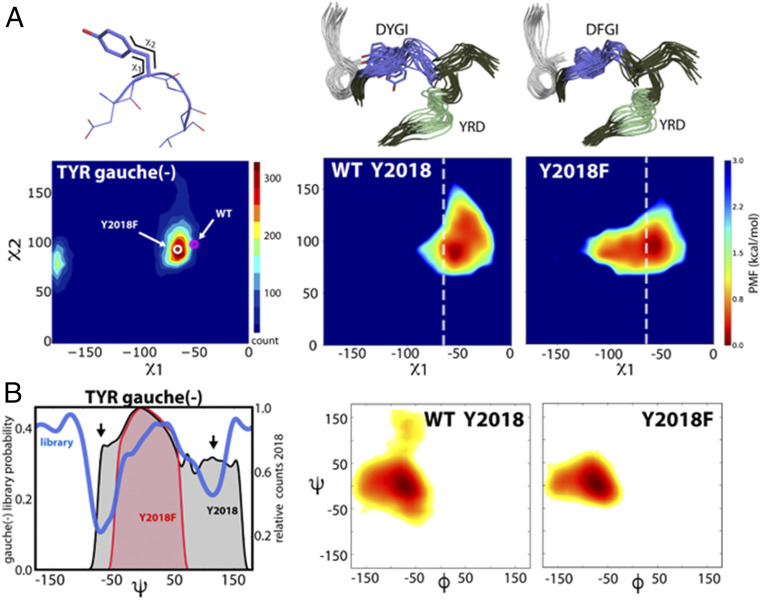
Y2018 introduces strain into the DYGI loop. (*A*) The WT Y2018 side chain is in a high-energy conformation (magenta circle), illustrated by the side-chain torsion of Y2018 overlaid with the ideal tyrosine χ1/χ2 gauche(-) torsions (45), shown with increasing torsion preference (blue to red), whereas Y2018F (white circle) relieves the nonideal conformation. The χ1/χ2 side-chain torsion angle distribution for Y2018 and Y2018F during the simulations demonstrates the WT deviation from ideal space (*Right*). The white dashed lines indicate the preferred χ1 for Tyr. (*B*) The consequence of the nonpreferred side-chain conformation in WT manifests as strain in the backbone conformation, illustrated by the probability distribution of the Tyr ψ-dihedral angle. The ideal backbone conformation for the Tyr gauche(-) rotamer (45) is depicted in blue. The nonpreferred Y2018 rotamer in WT adds strain to the backbone (black area; arrows indicate nonideal backbone conformations). Y2018F populates ideal dihedral space (red area). The φ/ψ distribution of the Y/F2018 backbone during the simulation is shown (*Right*). The WT Y2018 backbone is more disordered than Y2018F. The mutation stabilizes the entire DYGI backbone as also illustrated in the conformational ensemble from MD (*A*).

The hydroxyl moiety of Y2018 in the DYGI motif forms persistent hydrogen bonds between the backbone of both I1933 in the αC-β4-loop and I2015 (Fig. 7*B*). This interaction stabilizes the tyrosine side chain in an orientation that restricts the αC-helix from assembling the active site due to steric clash with L1924. This simulation agrees very well with a recent cryoelectron microscopy structure of an inactive conformation of LRRK2_RCKW_ (5). These authors identify the same hydrogen bond between Y2018 and the shell residue I1933. Our simulations provide strong independent evidence that Y2018 in WT LRRK2 is a key stabilizer of the inactive kinase conformation and may also act as a sensor of the αC-β4-loop conformation, a conserved hotspot for kinase allosteric modulation (26). Absence of the OH hydrogen bonds in the Y2018F mutation leads to greater Y2018F side-chain dynamics and packing with L1924 that resemble an active kinase configuration (or a properly formed regulatory spine; R spine). An assembled R spine is the hallmark for an active kinase. Stabilization of the WT Y2018 side chain leads to a strained DYGI motif and “frustrated” DYGI backbone free-energy landscape by pulling the motif out of ideal ϕ/ψ space, which is likely important for the kinase’s role as a switch (Fig. 8). A consequence of freeing the side chain of Y2018F is the convergence of the DYG dihedral angles into a canonically active kinase ϕ/ψ region (27) (Fig. 8 and *SI Appendix*, Fig. S4).

The I2020T mutation introduces a hydrogen bond between the OH group of the threonine and the backbone of the catalytic YRD motif (Y1992) (Fig. 7*C*). The interaction with the YRD motif both stabilizes the catalytic loop and also leads to a closed kinase active site (Fig. 7 *A* and *C*). The DYGI motif is also stabilized in an active conformation, similar to Y2018F, as measured by its ensemble DYG dihedral angles (*SI Appendix*, Fig. S4). The I2020T equilibrium is shifted to the closed conformation and activity may be reduced because the mechanism for opening is impaired. Finally, G2019S introduces a hydrogen bond with the side chain of E1920 in the αC-helix, which in turn forms a highly conserved salt bridge with K1906 of β3 (Fig. 7*D*). The influence of the G2019S mutation on the interaction between αC and β3 and the DYGI loop favors the closed and active kinase conformation. The G2019S DYGI motif is also stabilized in an active conformation as described by its dihedrals (*SI Appendix*, Fig. S4).

## Discussion

The detailed signaling cascades that control LRRK2 are still being elucidated, and the molecular mechanisms that control its intrinsic regulation are also not well-characterized. Here we investigated a four-domain construct of LRRK2 consisting of the ROC, COR, kinase, and WD40 domains, which is the shortest functional construct to date that retains kinase as well as GTPase activity and is also the smallest construct that can dock onto MTs (5). In the current work, we elucidate different aspects of the intrinsic regulation of LRRK2 using a multilayered approach focusing on the importance of the kinase domain. We first concentrated on the spatial and temporal distribution of full-length LRRK2 in cells as a function of the high-affinity kinase inhibitor MLi-2, which provided us with a real-time assay for reversible filament formation in live cells. The effects of removing the N-terminal targeting domains on cellular distribution were then explored with our LRRK2_RCKW_ variants, which led us to predict that NTDs shield and inhibit the catalytic domains when LRRK2 is in its inactive resting state. Biochemical characterization of LRRK2_RCKW_ variants demonstrated that substrate-specific kinase activity comparable to full-length LRRK2 was retained by LRRK2_RCKW_; the catalytic machinery for mediating phosphoryl transfer remained intact. We next used HDX-MS analysis of LRRK2_RCKW_ to provide a portrait of the conformational states of LRRK2_RCKW_ in the presence and absence of MLi-2. Mapping the solvent-accessible regions in a model of the LRRK2 kinase domain not only provides an allosteric portrait of the “breathing” kinase domain but also suggests multidomain cross-talk in LRRK2_RCKW_. Finally, we performed GaMD calculations on the LRRK2 kinase domain to elucidate at a molecular level the differences in breathing dynamics between WT LRRK2 and the pathogenic kinase domain mutations Y2018F, G2019S, and I2020T, explicitly establishing the role of the DYGI motif as a dynamic regulator of the switch mechanism. With this multiscale approach, we were able to clearly demonstrate that the kinase activity and the spatial distribution of LRRK2 are regulated by a complex interplay of all the embedded protein domains. The highly dynamic kinase domain, nevertheless, appears to be the driver that coordinates the overall domain cross-talk and serves as a central regulatory hub for the intrinsic regulation of LRRK2.

### Filament Formation Is Dependent on Unleashing the Catalytic Domains and on the Conformation of the Kinase Domain.

Although multiple functions are associated with the many domains of LRRK2, these domains can be structurally and functionally divided into the catalytically inert NTDs and the catalytic CTDs, and distinct functions are embedded in each. Further complexity is introduced by heterologous proteins such as Rab GTPases and 14-3-3 proteins, which also contribute to the activation and subcellular localization of LRRK2. LRRK2 also exists in multiple oligomeric states where the most active state is thought to be a dimer in contrast to the less active monomer (19). The stability of the monomers and dimers can be further facilitated by heterologous proteins, in particular the 14-3-3 proteins. Physiologically, LRRK2 is thought to be activated by Rab GTPases, such as Rab29, which dock onto the NTDs and target LRRK2 to the *trans*-Golgi network (14). Other Rabs may also activate LRRK2 and target it to different organelles (13) while autophosphorylation on residues such as S1292 likely is a subsequent step in the activation process (5, 28). Many of the PD mutations hijack these finely tuned regulatory mechanisms. Constitutive localization to MTs is a phenotype displayed by three of the four common LRRK2 PD mutants (R1441C, Y1699C, and I2020T) while the hyperactive G2019S mutant, like WT LRRK2, remains cytosolic. Using cryoelectron tomography (cryo-ET), Watanabe and coworkers (25) were able to capture the precise way in which this LRRK2 mutant (I2020T) can polymerize and dock onto MTs. They show specifically how this LRRK2 mutant forms periodically repeating dimers, which then polymerize in a helical array onto MTs. The cellular phenotypes associated with G2019S, I2020T, and R1441C/Y1699C and other PD-associated mutations include perturbation of MT-related processes such as vesicular trafficking, autophagy, cilia formation, and nuclear/mitochondria morphology, so it is very likely that LRRK2 dysfunction physiologically interferes globally with dynamic cross-talk with MTs (9, 29–31).

With live-cell imaging in the absence and presence of a kinase inhibitor, MLi-2, we were able to capture at low resolution in real time the relocalization of cytoplasmic WT and G2019S LRRK2 to decorated MTs, a process that is fully reversible when the inhibitor is removed. MLi-2 binding to WT and G2019S thus captures the pathogenic phenotype constitutively observed for I2020T, R1441C, or Y1699C. In contrast to MLi-2 and LRRK2-IN-1, which are both type I kinase inhibitors, we show that in the presence of a type II kinase inhibitor, rebastinib, G2019S LRRK2 remained cytosolic, confirming the predictions of Deniston and coworkers that docking to MTs is extremely sensitive to the conformational state of the kinase domain (5). Although the MLi-2 complex is catalytically inactive, MLi-2, which is a competitive inhibitor of ATP, nevertheless locks the kinase into an active-like conformation (24). In contrast, rebastinib is likely to stabilize a DYG/DFG-out/open and inactive conformation of the kinase domain. In the absence of the NTDs, both WT and G2019S LRRK2_RCKW_ spontaneously form filaments independent of MLi-2. Our HDX-MS data confirm that deletion of the NTDs in LRRK2_RCKW_ does not unfold the remaining CTDs as solvent exchange shows that the protein is properly folded with the domains well-packed against each other. Collectively, our results support a model where the catalytically inert NTDs function as a lid that shields the active sites of the CTDs. The lid can be unleashed physiologically by activating Rab GTPases or by mutations that make LRRK2 a risk factor for PD. Two of the PD mutants, R1441C/G/H and Y1699C, are localized in the ROC and COR domains, respectively, and presumably disrupt a domain–domain interface which is sufficient to unleash the NTDs. While targeting, activation, and inhibition of the CTDs are important functions that are embedded in the NTDs, our biochemical studies here show that all of the kinase activity of full-length LRRK2 is embedded in LRRK2_RCKW_. The other two common PD mutations (G2019S and I2020T), although functionally distinct, are in the kinase domain. Our live-cell imaging together with our biochemical and simulation results, discussed below, and the recently published cryo-ET structure (25), all support the hypothesis that unleashing the NTD lid as well as an active conformation of the kinase domain, not necessarily kinase activity, are essential requirements for dimerization and MT binding.

### The Switch Mechanism for Activation of LRRK2 Is Embedded in the DYGI Motif of the Kinase Domain.

With HDX-MS, we confirm that a shift in conformation is induced by the binding of MLi-2 to LRRK2_RCKW_ and, significantly, we find that stabilizing the active kinase conformation with MLi-2 drove changes in conformation and domain organization throughout LRRK2_RCKW_. The global decrease in backbone deuterium exchange measured across all four domains particularly in the linker between the COR-B and kinase domains and in flexible regions throughout LRRK2_RCKW_ (Fig. 5*A*) is suggestive of changes in domain–domain packing as well as changes in global conformational dynamics. We propose that changes in the CTD organization and dynamics, driven by stabilization of the active kinase conformation, are likely coupled with association and dissociation of the NTDs. This explains why mutations such as R1441C and Y1699C, that lie far from the kinase active site but at a domain interface, are capable of unleashing the NTDs. Using the MLi-2–bound LRRK2_RCKW_ as a proxy for the frozen closed and active-like conformation of the kinase domain, we explored each of the activating DYGI mutants using MD simulations and asked how each mutation perturbs the conformational ensemble of the kinase domain.

Our first hint that the DYGI motif impacts the kinase domain with consequences for LRRK2 global conformation and regulation came from our previous work with the DYGI mutations Y2018F and I2020T, where we were able to correlate changes in the internal organization of the kinase domain, specifically assembly of the R spine, with in situ MT association (10). Our data here show that deletion of the NTDs induces a similar MT association phenotype independent of mutation. Although full-length G2019S, the most prevalent PD mutation, does not form significant filaments spontaneously, we show that its kinetics of MT association are increased relative to WT following treatment with MLi-2 (Movies S1, S2, S3, and S4), suggesting that this mutation may share a common deregulating mechanism through changes in the kinase domain conformation. Indeed, our GaMD calculations reveal that in WT LRRK2 the DYGI motif exists in a strained conformation and that release of the strain leads to stabilization of the DYGI dynamics and promotion of the active kinase conformation by all three mutations Y2018F, G2019S, and I2020T. However, each of the mutants releases the strained DYGI motif and stabilizes the closed active conformation by different atomistic mechanisms. Significantly, the WT kinase in these simulations still fluctuates between both inactive and active states while favoring the inactive conformation. This means input from external factors, such as the NTDs or heterologous proteins, is required to fully modulate the conformational equilibrium to inhibit kinase activity. Activating DYGI mutations subvert the built-in regulation by distorting the inherent conformational equilibrium to a degree that breaks these layers of control. On the other hand, the kinase-dead D2017A, which has significantly reduced localization to MTs even in the absence of the NTDs, cannot be stabilized in an active-like kinase conformation because its active-site cleft is further dynamically destabilized. Together with our findings for rebastinib and MLi-2, this further emphasizes that filament formation is not solely dependent on the inhibitory lid function of the NTD but also on kinase domain integrity and conformational state.

Our findings highlight that the dynamic activation of LRRK2 is accurately defined in terms of a shift in conformational ensemble and associated dynamics. This is illustrated in the MD simulations by changes in bulk conformations due to DYGψ mutants and also by changes in timescales of dynamics highlighted by live-cell imaging and by the induction of bimodal HDX kinetics after binding MLi-2. HDX shows that the activation loop peptide has two distinct conformational populations. One represents a minor population of a highly solvent-exposed species similar to what was seen in the apo state and an additional highly protected species, which gradually becomes more solvent-exposed (Fig. 6). In MD simulations the DYGψ-activating mutants mirror this behavior and shift the equilibrium toward an ordered and less solvent-accessible activation loop (*SI Appendix*, Fig. S5). MLi-2 binding appears to lead to a large decrease in the kinetics of the exchange between states, effectively trapping a major population of a closed and active-like state of the kinase domain. Extending this concept of regulation by tuning of conformational dynamics to the cellular level, our work implies that changes in the balance of LRRK2 conformational equilibrium will lead to proportional changes in its cellular distribution and activity, that is, the population of LRRK2 in the cytosol vs. associated with MTs will mirror the conformational distribution of expanded and closed LRRK2.

### LRRK2 and BRaf Share the Same Kinase Activation Mechanism.

**Fig. 9.**
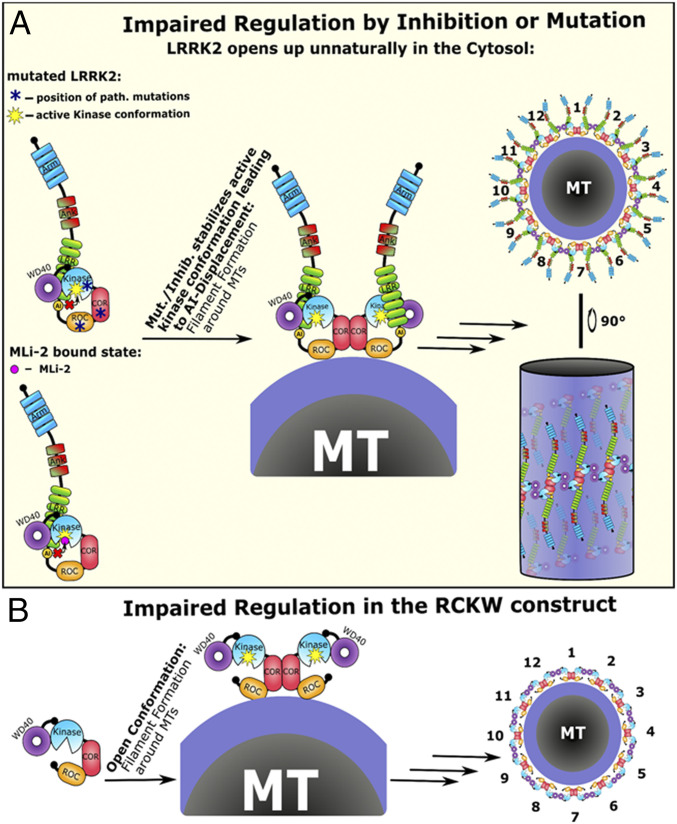
Impaired LRRK2 activation results in microtubule association. (*A*) In the mutated or MLi-2–inhibited state the autoinhibitory site and the NTDs get displaced already in the cytosol, and the kinase domain is in a constitutively active conformation. This short circuits the activation process which depends on Rab29 association. The active kinase conformation is sufficient to induce dimerization and thereby multimerization of LRRK2 around MTs. The LRRK2 multimers on the MT results in the filament formation phenotype. (*B*) The importance of the NTDs for stabilizing LRRK2 in an inactive cytosolically distributed state was tested by deleting the N terminus. The resulting LRRK2_RCKW_ deletion construct spontaneously forms filaments around MTs and is no longer able to become recruited by Rab29 as it lacks the interaction sites in the Arm and/or Ank domain. We believe that the missing AI domain, which we think is positioned in the NTDs, forces the kinase domain of the RCKW construct into an active conformation resulting in multimerization and docking to MTs.

By comparing the resulting finely tuned multilayered regulation mechanism of LRRK2 with other related homologs of the kinase tree, we recognized that our model for LRRK2 regulation closely resembles the activation process of another multidomain kinase: BRaf (32, 33). LRRK2, like BRaf, is activated by the interaction of its N-terminal noncatalytic domains with a small GTPase: Rab vs. Ras. In both cases, autoinhibitory sites/domains (AIs) in the NTDs become displaced when the activated GTPase binds, and this unleashes the kinase domain (34, 35). The kinase domains, no longer locked in their inactive conformations, are free to toggle between their inactive and active states. Only in the active conformation are the catalytic domains able to dimerize. In BRaf this pushes the kinase domain into the active conformation and induces *cis*-autophosphorylation of the activation loop. Dimerization of the catalytic domains of LRRK2, as seen in the cryo-ET structure (25), also requires an active conformation of the kinase domain, although in the case of LRRK2 the dimer interface most likely does not directly involve the kinase domain. In both cases this last step stabilizes the kinase domain in a conformation where the R spines are assembled, rendering them ready to bind and phosphorylate their substrates. When this process is disrupted by a mutation such as Y2018F or I2020T in the kinase domain of LRRK2, kinase activation becomes independent of Rab binding, as these mutations shift the equilibrium to a more active kinase conformation which also promotes displacement of the NTDs (Fig. 9*A*). This recapitulates a theme observed in BRaf, wherein the most activating cancer-driving mutation, V600F, renders the kinase active without the need for heterologous activation by Ras (36).

Other mutations that contribute to stabilization of the assembled R spine also lead to constitutive activation that is independent of Ras (36, 37). Kinase inhibition was also shown to facilitate downstream signaling as the inhibition of BRaf stabilized the active kinase conformation which is sufficient to promote *cis*-autophosphorylation through heterodimerization (38). This closely resembles the situation we observed for LRRK2 where MLi-2 stabilizes the active kinase conformation and thereby induces filament formation (Fig. 9*A*). The recently solved LRRK2 filament structure on MTs shows that each LRRK2 monomer interacts with two adjacent monomers through COR and the WD40 domain while the N terminus interacts with the upper and lower turns of the LRRK2 filament (25). Furthermore, the number of LRRK2 dimers needed for one turn correlates well with the number of MT protofilaments. Therefore, we believe that this is a pathogenic but specific interaction with MTs resulting from impaired regulation of LRRK2 by stabilizing an active conformation of the kinase domain of LRRK2 either by mutations or by binding of MLi-2.

Another feature common to LRRK2 and BRaf is that the deletion of the N terminus of either protein generates a constitutively active kinase (Fig. 9*B*) (39–41). Finally, the kinase-dead mutations in both proteins are unable to form productive dimers (42, 43). Since many aspects of the kinase domain regulation of LRRK2 and BRaf appear to be quite similar, it can be concluded that activation mechanisms, in general, where the kinase domain switching between active and inactive conformations serves as a central hub are likely to be conserved throughout much of the kinome. In addition, from the recent structures of full-length BRaf in complex with its substrate MEK and 14-3-3 proteins, we can see how these auxiliary proteins can stabilize either an inactive or an active conformation (44). This will certainly also be true for LRRK2. Clearly, we can learn much about LRRK2 activation by looking at close homologs that have been studied for a longer time, and hopefully this will facilitate the discovery of new therapeutic strategies for attacking LRRK2 as a driver of PD.

## Conclusion

Analysis of multidomain kinases suggests that the conformation of the kinase domain might, as a general principle, regulate much more than just the activity of the kinase. Our results with LRRK2 demonstrate that the active kinase conformation not only switches the kinase into an “on” state but also unleashes inhibitory domains, can promote dimerization, and can facilitate translocation to anchored substrates. In the case of LRRK2 this is a complex and tightly regulated process where more domains and other proteins than just the kinase domain are involved; however, the paradigm is similar for BRaf and probably for most kinases such as Src and PKC where the kinase domain is also embedded in multidomain proteins. In each case, the conformation of the kinase domain seems to play a crucial role in these intrinsic regulatory processes. We also further confirm here how a switch mechanism for activation, embedded in the DFGψ motif of the kinase core, allows the kinase to toggle between inactive and active conformations which are then communicated to all parts of the protein. We also demonstrate here that the noncatalytic NTDs play an important regulatory role by shielding the catalytic CTDs in the absence of physiological activators. Interacting proteins like 14-3-3 or Rab proteins are likely to further fine-tune this regulation either positively or negatively by stabilizing certain conformations of LRRK2. This precisely controlled signaling process can also be hijacked by a variety of disease-driving mutations such as those that lead to PD.

## Materials and Methods

Detailed materials and methods are included in *SI Appendix*, *Materials and Methods*. LRRK2_RCKW_ proteins were expressed and purified from Sf9 or HEK293T cells. All Sf9 proteins that were used for HDX-MS were monodisperse and monomeric based on size-exclusion chromatography (*SI Appendix*, Fig. S6). Phosphorylation of Rab8a was measured by Western blotting using a pT72-specific antibody (MJF-R20; abcam; ab231706) and anti-His antibody (GE Healthcare; mouse). The kinase activity of the LRRK2_RCKW_ variants was measured by a microfluidic mobility-shift kinase assay using LRRKtide (RLGRDKYKTLRQIRQ-amide; GeneCust) as substrate. The AMBER16 package was used for GaMD simulations.

## Supplementary Material

Supplementary File

Supplementary File

Supplementary File

Supplementary File

Supplementary File

## Data Availability

All study data are included in the article and/or supporting information.
