# Supplementary material for: Conformation and dynamics of the kinase domain drive subcellular location and activation of LRRK2
Source: Proc Natl Acad Sci U S A. 2021 Jun 4;118(23):e2100844118. doi: 10.1073/pnas.2100844118 (PMC8201809; doi:10.1073/pnas.2100844118)
Supplement: Supplementary File [file pnas.2100844118.sapp.pdf]

SUPPORTING INFORMATION:

MATERIAL AND METHOD

HEK293T Cell culture and Transfection

For expression of each Flag-Strep-Strep-tagged (FSS) $LRRK2_{RCKW}$ construct cells on ten 15 cm Ø cell culture dishes were transfected. Therefore, $1.0x10^7$ HEK293T cells (human embryonic kidney cells carrying the temp sensitive mutant of the SV-40 large T-antigen, *DSMZ*, DSMZ-No:ACC635) were seeded per dish and incubated for 24 h at 37°C and 5% CO_2 in Dulbecco's Modified Eagle Medium (DMEM) high glucose (w. L-Glutamine; w.o. Sodium Pyruvate, *biowest*) supplemented with 10% fetal bovine serum (FBS). In the following, transfections were performed by adding a 30 min preincubated mixture of 15 µg plasmid DNA (pCDNA3.0-FSS-$LRRK2_{RCKW}$ (aa1327-2527); NM_198578), 150 µl PEI (Polyethylenimine) (1 µg/µl) and 1.5 ml DMEM high glucose per dish. Medium was exchanged with fresh DMEM (high glucose, +10% FBS) after 24 h. After another 24 h cells were harvested and stored at -20°C before use.

$LRRK2_{RCKW}$ Transfection and Expression in Sf9 cells

Exponentially growing TriEx Sf9 insect cells (cells are derived from a high-yielding clone of *Spodoptera frugiperda* cell IPLB Sf21-AE (ATCC CRL-1711), *Novagen*, Prod.-No.: 71023-3) were diluted to a density of $2x10^7$ cells/mL. To initiate $LRRK2_{RCKW}$ expression, a high-titer virus suspension was added in the ratio 1:64. The viruses had been generated using the Bac-to-Bac expression system (*Invitrogen*) and the expression vector pFB-6HZB. Accordingly, the expressed protein was composed of an N-terminal His_6-Z tag and the LRRK2 residues 1327 to 2527. The infected cells were incubated in 800 mL aliquots in shaker flasks (66 h, gentle agitation, 27°C), harvested by centrifugation and stored at -20°C.

Purification of overexpressed Flag-Strep-Strep-tagged $LRRK2_{RCKW}$ constructs from HEK293T Cells

Each pellet was resuspended in 10 ml of fresh ice cold lysis buffer (25 mM Tris-HCl pH7.5, 150 mM NaCl, 10 mM $MgCl_2$, 0.5% Tween 20, 500 µM GTP, cOmplete™ EDTA free protease inhibitor cocktail [*Roche*], PhosSTOP™ [*Roche*]) and incubated for 30 min at 4°C on a rotating wheel to lyse cells. In the following, cell debris were removed by centrifugation at 42,000 xg and 4°C for 40 min and a filtration step (0.45 µm sterile filter). The supernatant was loaded onto a Streptactin Superflow column (0.5 mL bed volume, *IBA Goettingen*) and purification was performed according to the manufacturer's protocol while all buffers were additionally supplemented with 500 µM GTP (*Biolog Life Science Institute*) and 10 mM $MgCl_2$. The purified $LRRK2_{RCKW}$ constructs were stored at -80°C containing 10% Glycerol and 0.5 mM TCEP. The $LRRK2_{RCKW}$ construct concentrations were determined after Bradford (1).

Purification of overexpressed $LRRK2_{RCKW}$ constructs from Sf9 cells

The $LRRK2_{RCKW}$ expression construct contained an N-terminal His_6-Ztag and a TEV protease cleavage site. For purification, the Sf9 cell pellets were washed with PBS, resuspended in lysis buffer (50 mM HEPES pH 7.4, 500 mM NaCl, 20 mM imidazole, 0.5 mM TCEP, 5% glycerol, 5 mM $MgCl_2$, 20 µM GDP) and lysed by sonication. The lysate was cleared by centrifugation and loaded onto a Ni NTA column. After vigorous rinsing with lysis buffer the His_6-Ztagged protein was eluted in lysis buffer containing 300 mM imidazole. Immediately thereafter, the eluate was diluted with a buffer containing no NaCl, in order to reduce the NaCl-concentration to 250 mM and loaded onto an SP sepharose column. His_6-ZTEV-$LRRK2_{RCKW}$ was eluted with a 250 mM to 2.5 M NaCl gradient and treated with TEV protease overnight to cleave the His_6-Ztag. Contaminating proteins, the cleaved tag, uncleaved His_6ZTEV-$LRRK2_{RCKW}$ and TEV protease were removed in another combined SP sepharose Ni NTA step. Finally, $LRRK2_{RCKW}$ was concentrated and subjected to gel filtration in storage buffer (20 mM HEPES pH 7.4, 800 mM NaCl, 0.5 mM TCEP, 5% glycerol, 2.5 mM $MgCl_2$, 20 µM GDP) using an AKTA Xpress system combined with an S200 gel filtration column.

Live Cell Imaging

Time-lapse imaging was conducted using an Olympus FluoView1000 laser scanning confocal microscope equipped with a CO_2 and temperature-controlled chamber (at 37°C) and a 60X 1.42 NA objective lens. The HEK293T cells (*CCLV*, RRID:CVCL_0063) were imaged in HBSS supplemented with 5% fetal bovine serum and 10 mM HEPES and maintained at 37°C throughout the experiment. YFP fluorescence and the differential interference contrast were collected, 515 nm excitation/530–630 nm emission. Images were recorded every 5 to 11 min for a total duration of 2 h or 14 h as specified in figure legends, sampling speed 2 µs/pixel, image size 640X640 pixels in 15-20 z slices/area (step size 1 µm). Cells were imaged before and after treatment with inhibitors MLi-2 (100 nM) or rebastinib (100 nM). For washout experiments, cells were washed 5 times with imaging media, 2 min each, and then the imaging session was resumed using the same settings as before washout. Image processing such as 3D volume rendering and 4D movie generation was done using the Imaris Software (Bitplane AG, St. Paul, MN) and the Image J software (rsb.info.nih.gov/ij).

Immunofluorescence and laser confocal imaging

HEK293T cells were seeded onto 6-well dishes containing poly-D-lysine-coated glass coverslips or onto 35mm poly-D-lysine-coated glass bottom dishes (MatTek Corporation, Ashland, MA, USA). For HEK293T cell transfection, 1 µg of Flag-Strep-Strep-(FSS)-tagged $LRRK2_{RCKW}$ cDNA and Lipofectamine 2000 reagent (ThermoFisher Scientific, USA) were used according to the manufacturer's protocol. After incubation for 48 h at 37 °C cells were treated for 2 h with the LRRK2 inhibitor MLi-2. Subsequently cells were fixed with 4% paraformaldehyde in phosphate-buffered saline (PBS) for 15 minutes at room temperature. Cells were washed in PBS, permeabilized in 0.1% Triton X-100, and blocked in 1% BSA,

50 mM glycine and 2% normal donkey serum. A rabbit anti-Flag antibody (Abnova Company, Cat. No. PAB0900) was mixed 1:200 in blocking buffer diluted five-fold in PBS. The primary antibody solution was applied to the cells for 1 h at room temperature. The secondary antibody (donkey-anti-rabbit-Alexa568, Invitrogen, Cat. No. A10042) was also diluted (1:100) in the blocking buffer diluted five-fold in PBS and applied for 1 h at room temperature. Samples were mounted with the antifade agent ProLong Gold with DAPI (ThermoFisher Scientific, USA). The Olympus Fluoview 1000 laser scanning confocal microscope utilizing a 60X oil immersion objective lens with a numerical aperture of 1.42 was used for confocal imaging. Z-stack images were acquired with a step size of 0.3 microns and processed using the Fiji software package (2). Cells expressing the different mutants were assessed for the presence of clear filamentous structures and quantified in two independent experiments in case of the untreated cells and in one experiment for the MLi-2 treated cells.

Rab8a phosphorylation by LRRK2$_{RCKW}$ variants and pathogenic mutations

Phosphorylation of T72 of Rab8a was measured via Western Blotting using a pT72 specific antibody. Prior to the blotting step onto a nitrocellulose membrane an *in vitro* kinase assay using kinase buffer (25 mM TRIS/HCl, pH 7.5, 50 mM NaCl, 10 mM $MgCl_2$, 1 mM ATP, 0.5 mM GTP, 0.1 mg/ml BSA and 1 mM DTT) and an SDS-PAGE were performed. For the kinase assay 2.5 µM (6xHis)-Rab8a (aa 6-175) were used as substrate for 200 nM of the LRRK2$_{RCKW}$ variants. Rab8a was phosphorylated at 30 °C for 7 min at 650 rpm on a shaker. To stop the reaction 1xNu-PAGE LDS sample buffer (*Invitrogen*, Cat. No. NP0007) supplemented with 250 µM DTT was added followed by an incubation at 80 °C for 5-10 min. After SDS-PAGE and western blotting, membranes were blocked with 5% (w/V) BSA in TBS-T (1x Tris-buffered saline supplemented with 0.1% Tween20) for 1 h. Subsequently they were incubated overnight at 4 °C with the primary antibodies against pT72 (MJF-R20, *abcam*, Cat. No. ab231706) and the His-tag of Rab8a (anti-His-Antibody, *GE Healthcare, mouse*). Both were diluted (1:1000) in blocking buffer. Membranes were then washed three times with TBS-T. After secondary antibody incubation (anti-rabbit IRDye800 and anti-mouse IRDye680, 1:15000, *LiCOR*) for 1h at RT signals were detected using the Odyssey FC imaging system (*LiCOR*).

Microfluidic Mobility Shift Kinase Assay (MMSKA)

To quantify the kinase activities of the LRRK2$_{RCKW}$ variants MMSKA were performed using 1 mM ATP and 1 mM LRRKtide (RLGRDKYKTLRQIRQ-amide, *GeneCust*) as substrates. For these assays two stock solutions were prepared: a 2x concentrated (conc.) LRRKtide solution (1900 µM LRRKtide, 100 µM Fluorescein-LRRKtide, 2 mM ATP) and a 2x conc. LRRK2$_{RCKW}$ variant solution (100-200 nM LRRK2$_{RCKW}$ variant, 20 mM $MgCl_2$, 1 mM GTP). All stock solutions were prepared using kinase buffer (25 mM TRIS/HCl, pH 7.5, 50 mM NaCl, 0.1 mg/ml BSA and 1 mM DTT). The reactions were started by mixing

both solutions in a 1:1 ratio in 384 well plates. Reactions were performed at 30 °C and monitored for 60-90 min using a LabChip EZ Reader (*PerkinElmer*). The slope (conversion rate, [m]=%/min) of the percental conversion plotted against the time was determined using a linear fit model of Graph Pad Prism 6 and was converted into a reaction velocity ([v_0]=µmol/min). Experiments for each mutant were performed at least in duplicates of duplicates for two independent LRRK2 expressions. Each dot represents the mean of a duplicate, while the dotted line represents the mean of the measured wt activity. To determine significant differences between $LRRK2_{RCKW}$ wt and mutant activity a one-way ANOVA test based on the Dunnett's multiple comparisons test was performed.

Titration assays using the high-affinity inhibitor MLi-2 (Merck, USA) were performed to determine the active protein concentrations of the $LRRK2_{RCKW}$ variants. Therefore, 24 µL of Buffer A (25 mM TRIS/HCl, 50 mM NaCl, 20 mM $MgCl_2$, 1 mM GTP, 1 mM DTT, 0.5 mg/ml BSA, 52.1/104.2 nM $LRRK2_{RCKW}$ variant) were mixed with 1 µL of an MLi-2 dilution series (50x concentrated) prepared in 100% DMSO. To start the reaction 10 µL of this reaction mix was added to 10 µL of Buffer B (25 mM TRIS/HCl, 50 mM NaCl, 1 mM DTT, 0.5 mg/ml BSA, 1900 µM LRRKtide, 100 µM Fluorescein-LRRKtide, 360 µM ATP). The resulting conversion rates were plotted against the respective MLi-2 concentrations and to obtain the active protein concentrations the x-axes intersection of the respective linear fit was determined using Graph Pad Prism 6 (assuming a 1:1 binding of MLi-2).

Hydrogen-deuterium exchange mass spectrometry

Hydrogen/deuterium exchange mass spectrometry (HDX-MS) was performed using a Waters Synapt G2Si equipped with nanoACQUITY UPLC system with H/DX technology and a LEAP autosampler. The sample concentration was 5 µM in LRRK2 buffer containing: 20 mM HEPES/NaOH pH 7.4, 800 mM NaCl, 0.5 mM TCEP, 5% Glycerol, 2.5 mM $MgCl_2$ and 20 µM GDP. The deuterium uptake was measured in LRRK2 buffer in the presence and absence of the kinase inhibitor MLi-2 (50 µM). For each deuteration time, 4 µL complex was equilibrated to 25 °C for 5 min and then mixed with 56 µL D_2O LRRK2 buffer for 0, 0.5, 1 or 2 min. The exchange was quenched with an equal volume of quench solution (3 M guanidine, 0.1% formic acid, pH 2.66). The quenched sample (50 µL) was injected into the sample loop, followed by digestion on an in-line pepsin column (immobilized pepsin, Pierce, Inc.) at 15 °C. The resulting peptides were captured on a BEH C18 Vanguard pre-column, separated by analytical chromatography (Acquity UPLC BEH C18, 1.7 µM, 1.0 X 50 mm, Waters Corporation) using a 7-85% acetonitrile gradient in 0.1% formic acid over 7.5 min, and electrosprayed into the Waters SYNAPT G2Si quadrupole time-of-flight mass spectrometer. The mass spectrometer was set to collect data in the Mobility, ESI+ mode; mass acquisition range of 200–2,000 (m/z); scan time 0.4 s. Continuous lock mass correction was accomplished with infusion of leu-enkephalin (m/z = 556.277) every 30 s (mass

accuracy of 1 ppm for calibration standard). For peptide identification, the mass spectrometer was set to collect data in MS^E, ESI+ mode instead.

The peptides were identified from triplicate MS^E analyses of 10 µM $LRRK2_{RCKW}$, and data were analyzed using PLGS 3.0 (Waters Corporation). Peptide masses were identified using a minimum number of 250 ion counts for low energy peptides and 50 ion counts for their fragment ions. The peptides identified in PLGS were then analyzed in DynamX 3.0 (Waters Corporation) using a cut-off score of 6.5, error tolerance of 5 ppm and requiring that the peptide be present in at least 2 of the 3 identification runs. The peptides reported on the coverage maps are those from which data were obtained. The relative deuterium uptake for each peptide was calculated by comparing the centroids of the mass envelopes of the deuterated samples vs. the undeuterated controls (3). For all HDX-MS data, at least 2 biological replicates were analyzed each with 3 technical replicates. Data are represented as mean values +/- SEM of 3 technical replicates due to processing software limitations, however the LEAP robot provides highly reproducible data for biological replicates. The deuterium uptake was corrected for back-exchange using a global back exchange correction factor (typically 25%) determined from the average percent exchange measured in disordered termini of various proteins (4). Deuterium uptake plots were generated in DECA (github.com/komiveslab/DECA) and the data are fitted with an exponential curve for ease of viewing (5).

Gaussian accelerated Molecular Dynamics (GaMD) simulation

The LRRK2 kinase domain constructs for simulations were prepared using Phyre2 (6) with the crystal structure of Src kinase (PDBID: 1Y57) serving as an initial template. The model was separately then mutated to Y2018F, G2019S, I2020T and phosphorylated at S2032 and T2035 to form the activated LRRK2 kinase (7, 8) and processed in Maestro (Schrodinger). The Protein Preparation Wizard was used to build missing sidechains and model charge states of ionizable residues at neutral pH. Hydrogens and counter ions were added and the models were solvated in a cubic box of TIP4P-EW water (9) and 150 mM KCl with a 10 Å buffer in AMBER tools D.A. Case, 2016 #731}. AMBER16 was used for energy minimization, heating, and equilibration steps, using the CPU code for minimization and heating and GPU code for equilibration. Parameters from the Bryce AMBER parameter database were used for phosphoserine and phosphothreonine (10). Systems were minimized by 1000 steps of hydrogen-only minimization, 2000 steps of solvent minimization, 2000 steps of ligand minimization, 2000 steps of side-chain minimization, and 5000 steps of all-atom minimization. Systems were heated from 0 K to 300 K linearly over 200 ps with 2 fs time-steps and 10.0 kcal mol Å position restraints on protein. Temperature was maintained by the Langevin thermostat. Constant pressure equilibration with an 8 Å non-bonded cut-off with particle mesh Ewald was performed with 300 ps of protein and peptide restraints followed by 900 ps of unrestrained equilibration. Gaussian accelerated MD (GaMD) was used

on GPU enabled AMBER16 to enhance conformational sampling (11). GaMD applies a Gaussian distributed boost energy to the potential energy surface to accelerate transitions between meta-stable states while allowing accurate reweighting with cumulant expansion. Both dihedral and total potential acceleration were used simultaneously. Potential statistics were collected for 2 ns followed by 2 ns of GaMD during which boost parameters were updated for each simulation. Each GaMD simulation was equilibrated for 10 ns. For each construct 10 independent replicates of 200 ns of GaMD simulation were run in the NVT ensemble, for an aggregate of 2.0 µs of accelerated MD. Potential energy surfaces were determined along each reaction coordinate using a cumulant expansion to the second order.

SUPPLEMENTARY FIGURES

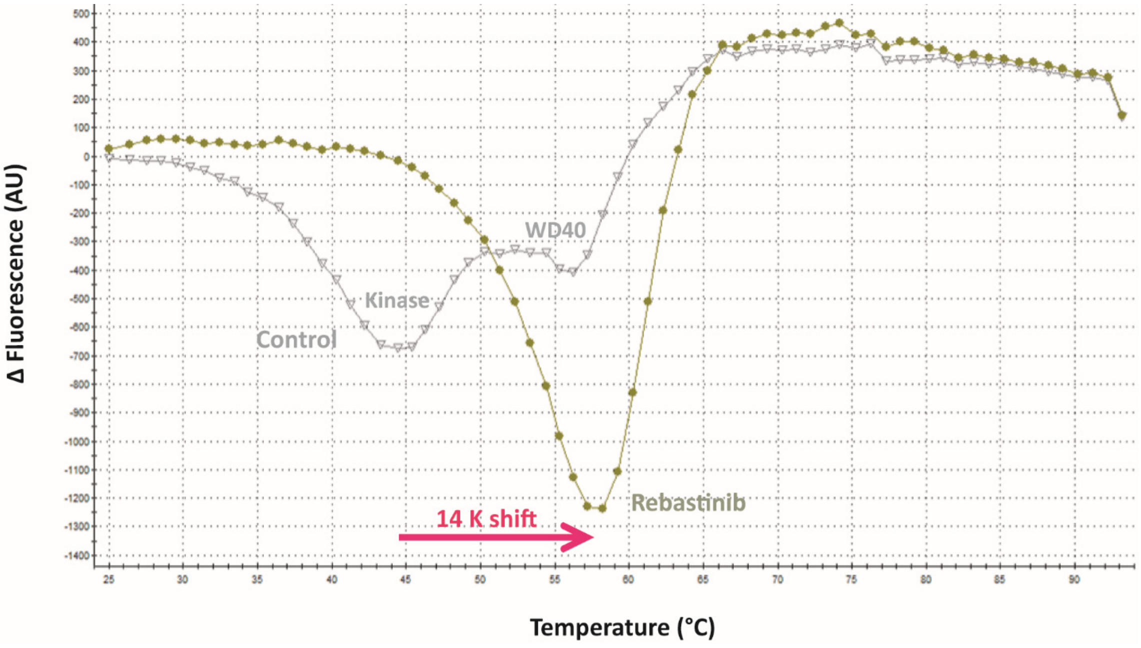

Figure S1. The type-2 kinase inhibitor rebastinib binds to the LRRK2 kinase domain. Shown are the 1st derivatives of the LRRK2 melting curves with or without rebastinib. The LRRK2 protein comprised two domains (kinase and WD40). Accordingly, there were two minima in the control curve. The addition of rebastinib stabilized the LRRK2 kinase domain and shifted its melting temperature by 14 K, indicating a binding constant <1 uM.

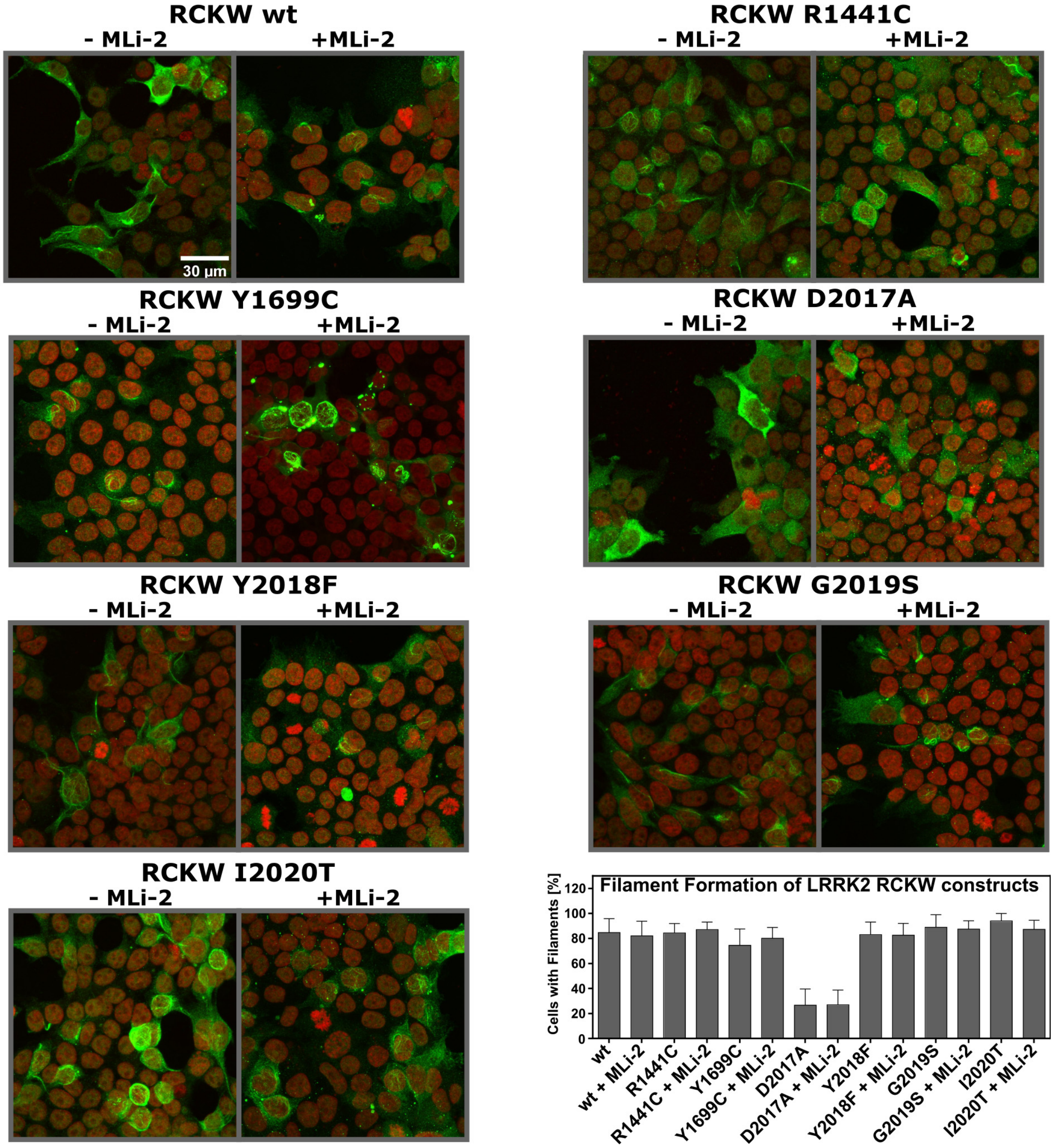

Figure S2. The localization of the LRRK2-G2019S mutant and $LRRK2_{RCKW}$ variants. All tested $LRRK2_{RCKW}$ variants displayed a high likelihood (80-90%) to form filaments inside the HEK293T cells except for $LRRK2_{RCKW}$ D2017A (20-30%). Interestingly, in contrast to LRRK2 full length the percentage of cells showing filament formation was independent of MLi-2 treatment or a specific $LRRK2_{RCKW}$ mutation.

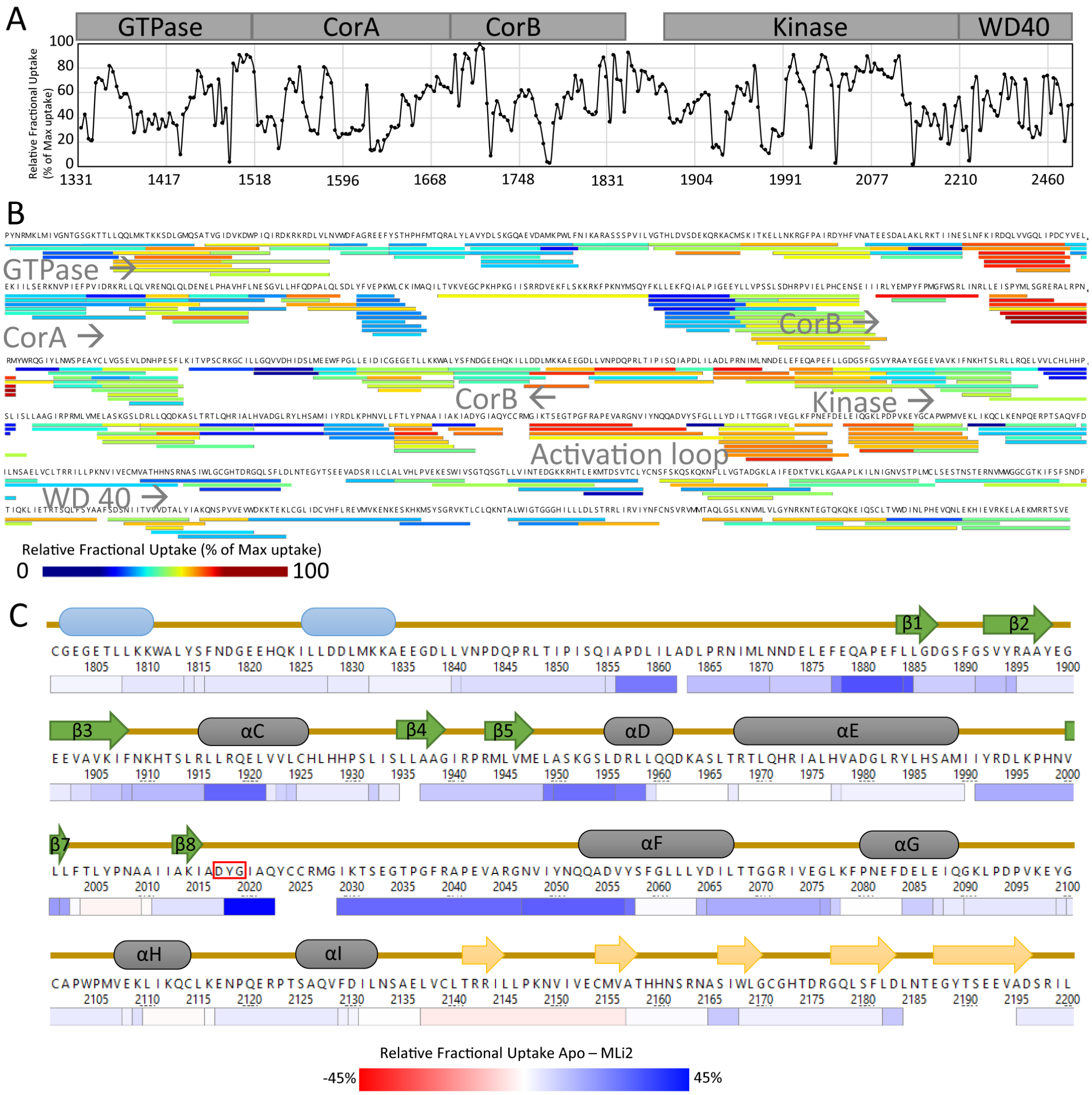

Figure S3. The identified HDX-MS peptides of LRRK2$_{RCKW}$. (A) The relative deuterium exchange for each peptide detected from the N to C terminus of LRRK2 at 2 min, apo condition. **(B)** Each line in the coverage map represents an identified peptide (exchanged 2 min) and the color indicates the relative deuterium uptake. Location of each domain is indicated. The map identifies folded regions as well as solvent exposed regions such as the activation loop in the kinase domain. The coverage is 96.3% and the redundancy is 3.72, which is successful for a protein this large. **(C)** The heat shows the relative fractional uptake by color at 2 min. It is colored based on the difference of relative deuterium uptake between apo and MLi-2 states.

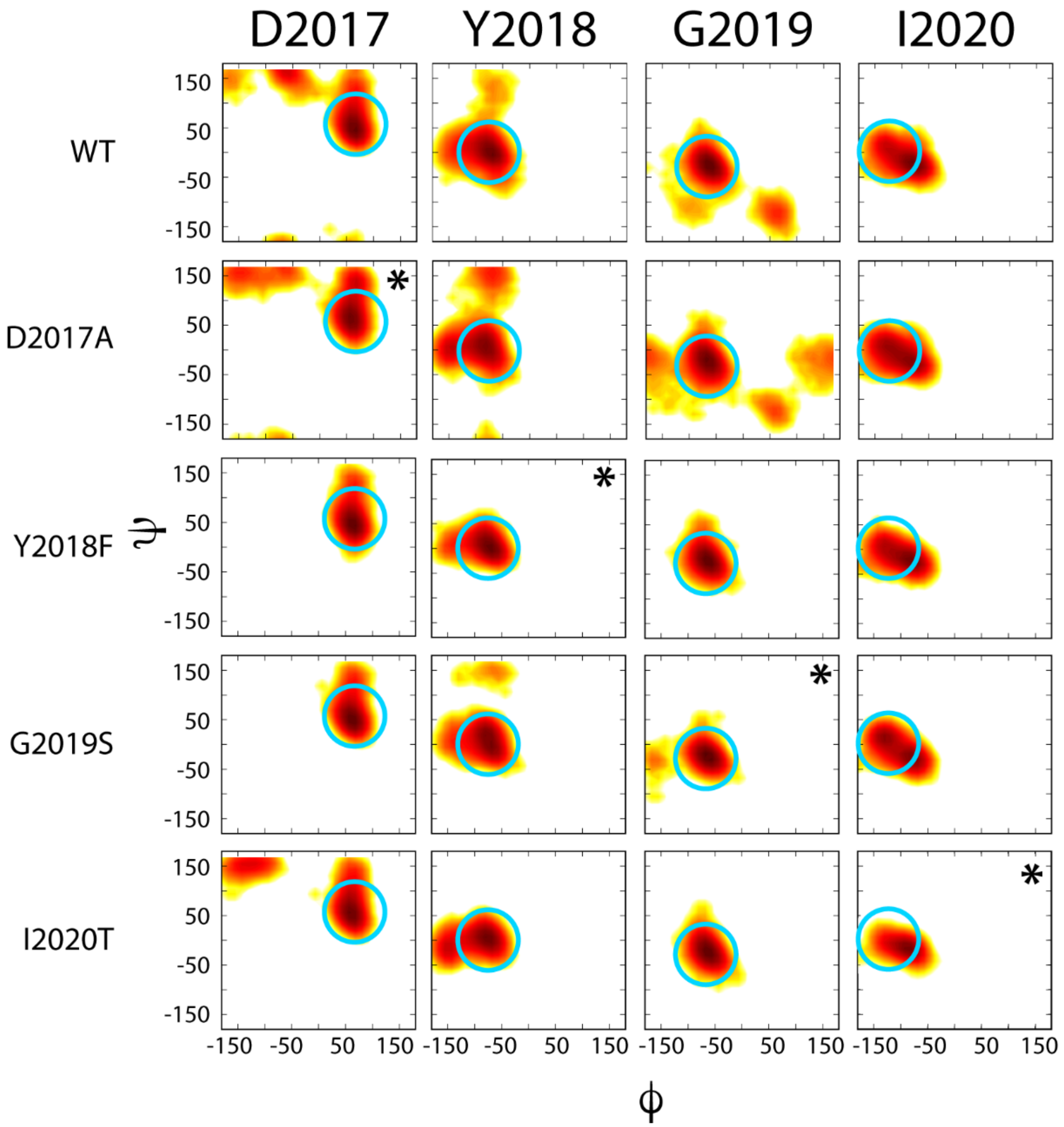

Figure S4. DYGI backbone dihedrals from MD simulations. DYGψ dihedral space that is associated with an activated kinase are circled in cyan (62). The wt kinase and D2017A mutant have a dynamic DYG loop that samples conformations associated with inactive kinases. The DYG loop of the mutants converge to a ϕ/ψ space that is typical of active / closed kinases. Asterisks indicate sites of mutation.

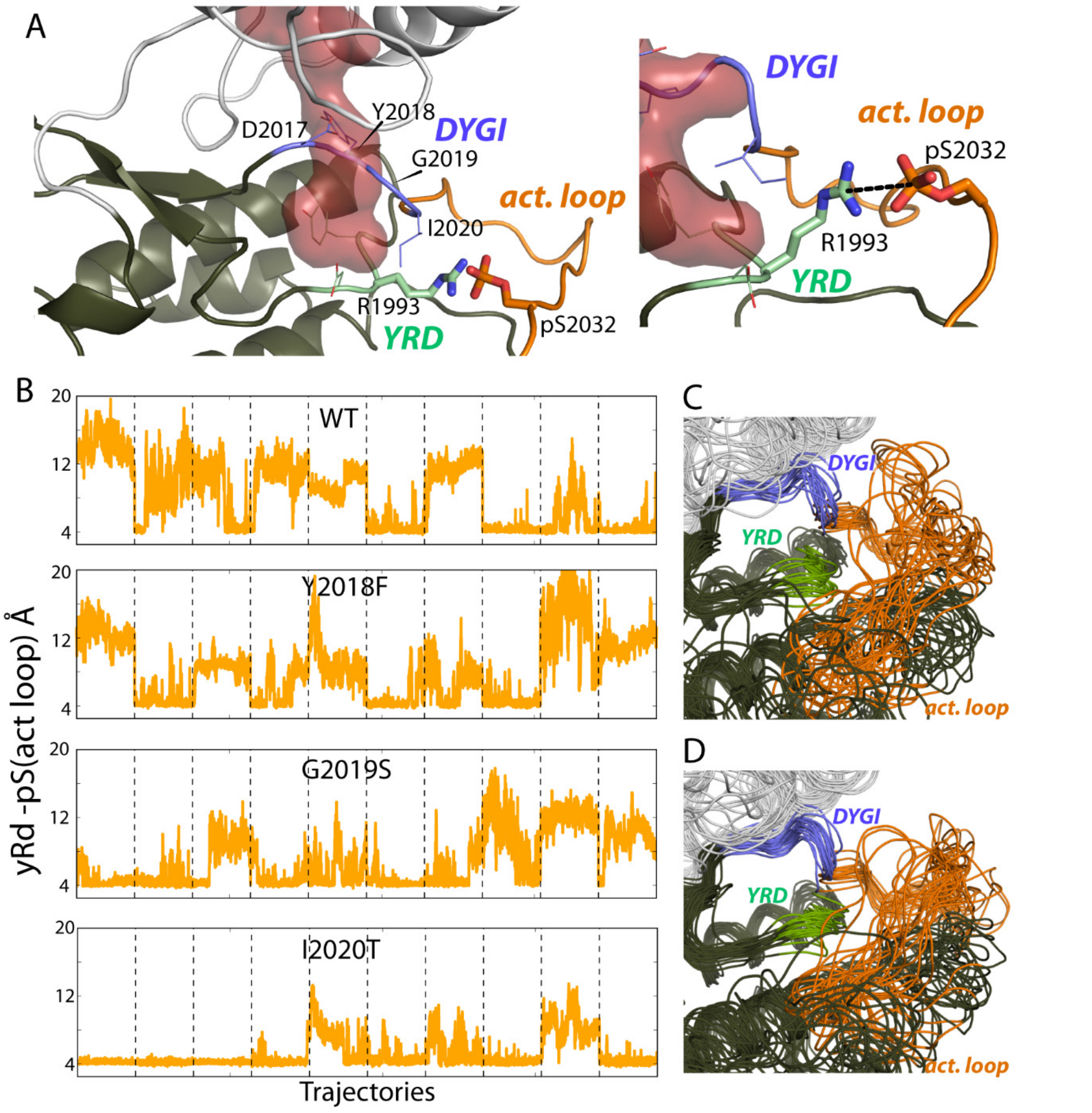

Figure S5. Activation loop stability from MD simulations. **(A/B)** The stability of the activation loop is represented as the distance between the YRD loop (R1993) and activation loop (pS2032). The salt-bridge between YRD and activation loop couples the catalytic loop with the N- and C-lobes. The activation loop of wt is the least stable, while activating DYGψ mutants increase stability. **(C)** The wt activation loop conformational ensemble is more widely distributed and more solvent accessible than **(D)** the I2020T activation loop. The DYGI motif is shown in blue, YRD motif in green, and activation loop in orange.

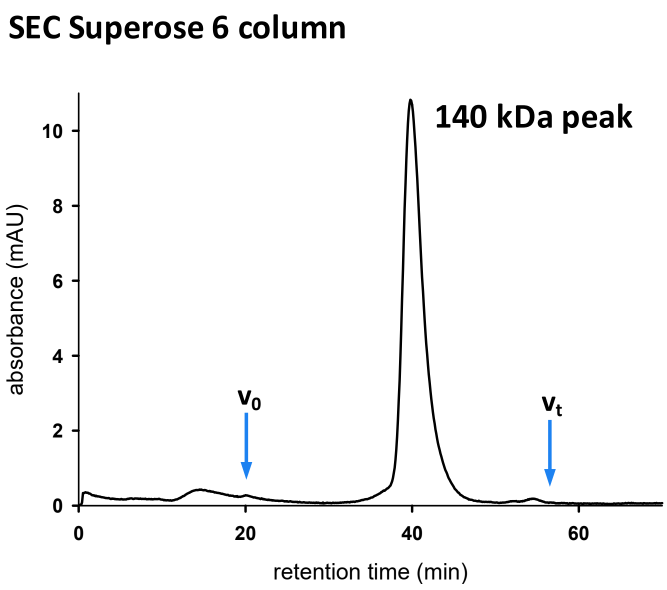

Figure S6. LRRK2$_{RCKW}$ is monodisperse in solution. In analytical SEC, the recombinant protein eluted in a single peak. The retention time 42 min indicated LRRK2$_{RCKW}$ to be monomeric, confirming earlier results by Deniston et al (12).

SUPPLEMENTARY VIDEOS:

Movie 1. Time-lapse imaging of HEK293T cells transiently expressing YFP-LRRK2-G2019S. The time interval is 5 minutes. MLi-2 was added right after the second frame. Images (640x640 pixels) of YFP fluorescence (515 nm excitation/530-630 nm emission) represent 3D volume reconstructions from confocal image stacks over time.

Movie 2. Time-lapse imaging of HEK293T cells transiently expressing YFP-LRRK2-G2019S following wash-out of MLi-2. The time interval is 11 minutes. Images (640x640 pixels) of YFP fluorescence (515 nm excitation/530-630 nm emission) represent 3D volume reconstructions from confocal image stacks over time.

Movie 3. Time-lapse imaging of HEK293T cells transiently expressing YFP-LRRK2-WT. The time interval is 5 minutes. MLi-2 was added right after the second frame. Images (640x640 pixels) of YFP fluorescence (515 nm excitation/530-630 nm emission) represent 3D volume reconstructions from confocal image stacks over time.

Movie 4. Time-lapse imaging of HEK293T cells transiently expressing YFP-LRRK2-WT following wash-out of MLi-2. The time interval is 11 minutes. Images (640x640 pixels) of YFP fluorescence (515 nm excitation/530-630 nm emission) represent 3D volume reconstructions from confocal image stacks over time.

SUPPLEMENTARY REFERENCE

1. Bradford MM (1976) A rapid and sensitive method for the quantitation of microgram quantities of protein utilizing the principle of protein-dye binding. *Analytical biochemistry* 72:248-254.
2. Schindelin J*, et al.* (2012) Fiji: an open-source platform for biological-image analysis. *Nat Methods* 9(7):676-682.
3. Wales TE, Fadgen KE, Gerhardt GC, & Engen JR (2008) High-speed and high-resolution UPLC separation at zero degrees Celsius. *Analytical chemistry* 80(17):6815-6820.
4. Ramsey KM, Dembinski HE, Chen W, Ricci CG, & Komives EA (2017) DNA and IkappaBalpha Both Induce Long-Range Conformational Changes in NFkappaB. *J Mol Biol* 429(7):999-1008.
5. Lumpkin RJ & Komives EA (2019) DECA, A Comprehensive, Automatic Post-processing Program for HDX-MS Data. *Molecular & cellular proteomics : MCP* 18(12):2516-2523.
6. Kelley LA, Mezulis S, Yates CM, Wass MN, & Sternberg MJ (2015) The Phyre2 web portal for protein modeling, prediction and analysis. *Nat Protoc* 10(6):845-858.
7. Greggio E*, et al.* (2008) The Parkinson disease-associated leucine-rich repeat kinase 2 (LRRK2) is a dimer that undergoes intramolecular autophosphorylation. *J Biol Chem* 283(24):16906-16914.
8. Li X, Moore DJ, Xiong Y, Dawson TM, & Dawson VL (2010) Reevaluation of phosphorylation sites in the Parkinson disease-associated leucine-rich repeat kinase 2. *J Biol Chem* 285(38):29569-29576.
9. Horn HW*, et al.* (2004) Development of an improved four-site water model for biomolecular simulations: TIP4P-Ew. *The Journal of chemical physics* 120(20):9665-9678.
10. Homeyer N, Horn AH, Lanig H, & Sticht H (2006) AMBER force-field parameters for phosphorylated amino acids in different protonation states: phosphoserine, phosphothreonine, phosphotyrosine, and phosphohistidine. *Journal of molecular modeling* 12(3):281-289.
11. Miao Y, Feher VA, & McCammon JA (2015) Gaussian Accelerated Molecular Dynamics: Unconstrained Enhanced Sampling and Free Energy Calculation. *Journal of chemical theory and computation* 11(8):3584-3595.
12. Deniston CK*, et al.* (2020) Structure of LRRK2 in Parkinson's disease and model for microtubule interaction. *Nature*.
